# Tetrahydrofuran: MAK-Begründung, Nachtrag

**DOI:** 10.34865/mb10999d9_4ad

**Published:** 2024-12-23

**Authors:** Andrea Hartwig, MAK Commission

**Affiliations:** 1 Institut für Angewandte Biowissenschaften. Abteilung Lebensmittelchemie und Toxikologie. Karlsruher Institut für Technologie (KIT) Adenauerring 20a, Geb. 50.41 76131 Karlsruhe Deutschland; 2 Ständige Senatskommission zur Prüfung gesundheitsschädlicher Arbeitsstoffe. Deutsche Forschungsgemeinschaft, Kennedyallee 40, 53175 Bonn, Deutschland. Weitere Informationen: Ständige Senatskommission zur Prüfung gesundheitsschädlicher Arbeitsstoffe | DFG

**Keywords:** Tetrahydrofuran, Kanzerogenität, MAK-Wert, maximale Arbeitsplatzkonzentration, Spitzenbegrenzung, Entwicklungstoxizität, Hautresorption

## Abstract

The German Senate Commission for the Investigation of Health Hazards of Chemical Compounds in the Work Area (MAK Commission) summarized and re-evaluated the data for tetrahydrofuran [109-99-9] to derive an occupational exposure limit value (maximum concentration at the workplace, MAK value) considering all toxicological end points. Relevant studies were identified from a literature search and also unpublished study reports were used. Tetrahydrofuran is not irritating to the skin but causes serious eye irritation. The most sensitive effect of tetrahydrofuran in subchronic inhalation studies is a concentration dependent reduced ciliary beat frequency and morphological damage to ciliary cells of the nasal and tracheal mucous membranes at 100 ml/m^3^ (LOAEC) and above. Concentrations of 200 ml/m^3^ and above caused adverse liver effects in mice. Based on the LOAEC of 100 ml/m^3^ for effects on the respiratory tract, a NAEC was extrapolated and after taking into account the increased respiratory volume at the workplace (see List of MAK and BAT values, chapters I b and I c) a MAK value of 20 ml/m^3^ has been set. As the critical effect is local, Peak Limitation Category I has been assigned with an excursion factor of 2. In developmental toxicity studies concentrations of 5060 and 4934 ml tetrahydrofuran/m^3^ caused a reduction of body weight gain and a decreased degree of sternebral ossification in the rat foetus; in mice the number of live foetuses was reduced at 1800 ml/m^3^. Both effects occurred at maternally toxic concentrations and no teratogenic effects were observed. Therefore, tetrahydrofuran remains assigned to Pregnancy Risk Group C. Tetrahydrofuran is not genotoxic. Female B6C3F1 mice developed liver adenomas and carcinomas after long-term-inhalation via a CAR receptor-mediated mechanism leading to enzyme induction and cell proliferation. In comparison with phenobarbital, only a weak activation of the receptor could be demonstrated for tetrahydrofuran. Increased incidences of liver carcinomas were observed only in female mice of this susceptible strain at the highest concentration tested. This concentration already led to elevated mortality in male mice due to toxic effects. In male rats, long-term inhalation of tetrahydrofuran caused renal adenomas which occurred concurrently with a combination of α-2u-globulin nephropathy and chronic progressive nephropathy. Neither mechanism is considered to be relevant to humans. Therefore, tetrahydrofuran has no longer been classified in a Carcinogen Category. Tetrahydrofuran shows no sensitizing potential. Skin contact is expected to contribute significantly to systemic toxicity, thus tetrahydrofuran remains designated with “H”.

**Table:** 

**MAK-Wert (2023)**	**20 ml/m^3^ (ppm) ≙ 60 mg/m^3^**
**Spitzenbegrenzung (2023)**	**Kategorie I, Überschreitungsfaktor 2**
	
**Hautresorption (2002)**	**H**
**Sensibilisierende Wirkung **	**–**
**Krebserzeugende Wirkung **	**–**
**Fruchtschädigende Wirkung (1989)**	**Gruppe C**
**Keimzellmutagene Wirkung **	**–**
	
**BAT-Wert (2001)**	**2 mg Tetrahydrofuran/l Urin^a)^**
	
Synonyma	Butylenoxid Cyclotetramethylenoxid Diethylenoxid Furanidin Oxolan Tetramethylenoxid
Chemische Bezeichnung (IUPAC-Name)	Oxolan
CAS-Nr.	109-99-9
Formel	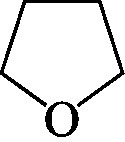
	C_4_H_8_O
Molmasse	72,11 g/mol
Schmelzpunkt	–108,44 °C (ECHA 2022)
Siedepunkt bei 1013 hPa	65 °C (ECHA 2022)
rel. Dichte bei 20 °C	0,883 g/cm^3^ (ECHA 2022)
Dampfdruck bei 20 °C	170 hPa (ECHA 2022)
log K_OW_	0,45 (ECHA 2022)
Löslichkeit	100 g/l Wasser (ECHA 2022)
**1 ml/m^3^ (ppm) ≙ 2,992 mg/m^3^**	**1 mg/m^3^ ≙ 0,334 ml/m^3^ (ppm)**
	
Hydrolysestabilität	k. A.
Verwendung	Lösungsmittel für Polyvinylchlorid, Vinylidenchloridpolymere, natürliche und synthetische Harze (insbesondere Vinyle) sowie in Deckbeschichtungslösungen, Polymerbeschichtungen, Cellophan, Schutzbeschichtungen, Klebstoffen, Magnetstreifen und Druckfarben, für Grignard- und Metallhydrid-Reaktionen, als Zwischenprodukt in der chemischen Synthese (ECHA 2022)

a) Stand 2024, wird reevaluiert

Zu Tetrahydrofuran liegt eine Begründung (Greim 2003) vor, in der alle früheren Bewertungen der Kommission zusammengefasst wurden. In diesem Nachtrag werden der MAK-Wert überprüft sowie neue Ergebnisse zum Wirkungsmechanismus und zu allen relevanten Endpunkten dargestellt. Zitierte unveröffentlichte toxikologische Studien von Firmen wurden der Kommission zur Verfügung gestellt.

## 1 Allgemeiner Wirkungscharakter

Tetrahydrofuran ist akut gering toxisch und wird inhalativ, oral und dermal gut resorbiert. Nach Aufnahme wird es in den Geweben gleichmäßig verteilt. Tetrahydrofuran wirkt nach akuter inhalativer Exposition bei Kaninchen ab 100 ml/m^3^ und bei Ratten nach subakuter und subchronischer Inhalation ab 100 bzw. 200 ml/m^3^ leicht reizend an der nasalen und trachealen Mukosa. Es treten verringerte Zilien-Schlagfrequenzen sowie morphologische Schäden der Zilien-tragenden Zellen auf. Wirkungen auf die Leber von Mensch und Ratte werden erst bei Konzentrationen um 1000 ml/m^3^ beobachtet. 

Unverdünntes Tetrahydrofuran führt an der Haut von Kaninchen zu leichten, an der Rattenhaut zu keinen Reizeffekten. Am Kaninchenauge werden irreversible Schäden an der Cornea hervorgerufen. 

Es liegen keine Hinweise auf eine allergene Wirkung von Tetrahydrofuran vor.

In Studien zur pränatalen Entwicklungstoxizität an Sprague-Dawley- und Crl:CD-Ratten treten bei gleichzeitiger Maternaltoxizität reduzierte Fetengewichte bei 5060 ml/m^3^ bzw. bei 4934 ml/m^3^ reduzierte Fetengewichte und eine verzögerte Ossifikation der Sternebrae auf. Bei Swiss-CD1-Mäusen ist bei 1800 ml/m^3^ die Anzahl lebender Feten pro Wurf bei gleichzeitiger maternaler Toxizität vermindert. Weder bei Ratten noch bei Mäusen kommt es zu teratogenen Effekten.

Tetrahydrofuran ist in mehreren Testsystemen in vitro und in vivo nicht genotoxisch. In Kurzzeittests hat Tetrahydrofuran weder ein zelltransformierendes noch ein tumorinitiierendes Potential. Tetrahydrofuran verursacht vermehrt Lebertumoren bei weiblichen B6C3F1-Mäusen und Nierenadenome bei männlichen F344-Ratten nach zweijähriger Exposition gegen 1800 ml/m^3^. 

## 2 Wirkungsmechanismus

Seit Erscheinen der Begründung im Jahr 2003 ist eine neue Untersuchung zum Wirkungsmechanismus von Choi et al. (2017) hinzugekommen. Zudem liegen neue Auswertungen (Bruner et al. 2010; Dekant 2019; Fenner-Crisp et al. 2011) zur Kanzerogenitätsstudie (NTP 1998) vor, die eine neue Bewertung des Wirkungsmechanismus erfordern. 

### 2.1 Reizwirkung

Die leicht reizende Wirkung von Tetrahydrofuran beruht vermutlich auf seiner entfettenden Wirkung aufgrund seiner Eigenschaften als Lösungsmittel.

### 2.2 Narkotische Wirkung

Die narkotische Wirkung könnte auf die im Metabolismus vermutlich aus Gammahydroxybuttersäure entstehende Gammaaminobuttersäure (GABA), einem bekannten neurotoxischen Stoff, zurückzuführen sein (Abschnitt 3.2).

### 2.3 Nierenadenome bei männlichen F344-Ratten

Alle vormals als Karzinome charakterisierten Neoplasien (Greim 2003; NTP 1998) in der Kanzerogenitätsstudie (NTP 1998) erwiesen sich in histopathologischen Nachuntersuchungen als Adenome (Bruner et al. 2010; Fenner-Crisp et al. 2011).

Für die Entstehung der Tumoren kann ein genotoxischer Mechanismus ausgeschlossen werden (Abschnitt 5.6).

Eine regenerative Zellproliferation aufgrund direkter Zytotoxizität wurde als möglicher Mechanismus der Tumorentstehung ausgeschlossen (Dekant 2019).

Aufgrund der Befunde der 2-Jahre-Kanzerogenitätsstudie (Abschnitt 5.7) und der mechanistischen Untersuchungen wurde eine Tumorentstehung durch eine α-2u-Globulin-vermittelte Nephropathie angenommen (Greim 2003). Als Bedingung eines derartigen Wirkungsmechanismus müssen laut IARC (Dybing und Sanner 1999) mehrere Kriterien erfüllt sein:

a) nachgewiesene nicht genotoxische Wirkung der Substanz oder deren Metaboliten
b) Nephropathie und renale Tumorbefunde nur bei männlichen Ratten
c) histopathologische Veränderungen mit Proteintröpfchen-Akkumulation in Kurzzeitstudien
d) Nachweis von α-2u-Globulin-Proteinakkumulationen in Tubulus-Zellen
e) reversible Bindung der Substanz oder eines ihrer Metaboliten an α-2u-Globulin
f) anhaltende erhöhte Zellproliferation im renalen Kortex
g) ähnliche Dosis-Wirkungs-Beziehungen sowohl bei Tumorbefunden als auch bei histopathologischen Endpunkten, die in Zusammenhang mit der α-2u-Globulin-Nephropathie stehen.

Für Tetrahydrofuran sind einige der Kriterien in den vorliegenden Studien mit Ratten jedoch nicht erfüllt:

Die Bindung von Tetrahydrofuran oder einer seiner Metaboliten an α-2u-Globulin ist nicht untersucht. Ein Zusammenhang der Neoplasien mit Protein- bzw. α-2u-Globulin-Akkumulation ist nicht eindeutig aufgezeigt worden, da Zahl und Größe der hyalinen Tröpfchen in der 14-Wochen-Studie des NTP nicht zunahmen. Weitere signifikante Anzeichen wie granuläre Zylinder von abgeschilferten Tubuluszellen an der Verbindungsstelle der äußeren und inneren Streifen der äußeren Medulla und eine lineare Mineralisation der papillären Tubuli in der 2-Jahre-Kanzerogenitätsstudie wurden nicht beobachtet. Eine Zellproliferation ist nicht untersucht worden (Fenner-Crisp et al. 2011; NTP 1998). Somit liegen nach erneuter Bewertung gemäß der Kriterien der IARC keine eindeutigen Belege für eine α-2u-Globulin vermittelte Nephropathie vor (BAuA 2017; Fenner-Crisp et al. 2011).

Ein weiterer möglicher Wirkungsmechanismus der Tumorentstehung ist die chronisch progressive Nephropathie (CPN). Histopathologische Belege für eine CPN sind Atrophie der Tubuli, Nierenvergrößerung und Verdickung der Basismembran des basophilen Tubulus. Ebenso treten hyaline Tröpfchen, Glomerulonekrose und interstitielle Fibrose auf. Eine fortgeschrittene CPN steht in Zusammenhang mit atypischen Tubulushyperplasien und Bildung renaler Tumore bei F344-Ratten (Hard et al. 2012). Die Ergebnisse der 2-Jahre-Kanzerogenitätsstudie des NTP und der histopathologischen Nachuntersuchungen liefern zwar deutliche Hinweise auf einen Zusammenhang von CPN und der Tumorentstehung, jedoch nimmt der Schweregrad der CPN bei den Tieren mit Adenomen durch Tetrahydrofuran nicht zu und die Inzidenz und Schwere der CPN bei Kontrolltieren und den Tieren der höchsten Konzentrationsgruppe waren ähnlich (Bruner et al. 2010; Dekant 2019; Fenner-Crisp et al. 2011). 

Trotz Limitierungen ergeben sich Hinweise auf Mechanismen über α-2u-Globulin und CPN. Es ist möglich, dass beide Prozesse bei der Entstehung der Nierenadenome zusammenwirken, sie besitzen jedoch beide keine Humanrelevanz. Die Autoren stellen eine nierenkanzerogene Wirkung von Tetrahydrofuran in Frage, da die Summe der als präneoplastisch angesehenen atypischen tubulären Hyperplasien und der Adenome in der Kontrollgruppe und in der höchsten Konzentrationsgruppe mit 6 bzw. 8 fast gleich war (Bruner et al. 2010). 

### 2.4 Lebertumoren bei weiblichen B6C3F1-Mäusen

Die Inzidenzen der Leberadenome und -karzinome waren bei der höchsten Konzentration von 1800 ml/m^3^ im Vergleich zur Kontrolle statistisch signifikant erhöht. Für beide Tumorarten zusammen lag die Inzidenz über dem Bereich der historischen Kontrollen (NTP 1998). 

Für die Entstehung der Tumoren kann ein genotoxischer Mechanismus ausgeschlossen werden (Abschnitt 5.6). Der zu Grunde liegende Mechanismus wurde in einer neu hinzugekommenen Studie von Choi et al. (2017) untersucht:

Je zehn weiblichen B6C3F1-Mäusen, C57BL/6-Mäusen und C57BL/6-Doppelknockout-Mäusen, defizient für den konstitutiven Androstanrezeptor und den Pregnan-X-Rezeptor (CAR/PXR) (C57BL/6-Nr1i2^tm3Arte^/Nr1i3^tm1.1Arte^), wurde mit einer Schlundsonde einmal täglich, sieben Tage lang, Tetrahydrofuran verabreicht. Alle Tiere waren sechs bis sieben Wochen alt. Die Dosen betrugen für B6C3F1-Mäuse 0, 300 und 1000 mg Tetrahydrofuran/kg KG und Tag und die hohe Dosis sollte der Konzentration entsprechen, die kanzerogen in der NTP-Studie (NTP 1998; siehe Abschnitt 5.7.2) war (umgerechnet 2300 mg/kg KG und Tag). Da jedoch die orale LD_50_ für Ratten bei 1650 mg/kg KG liegt, wurde die höchste Dosis auf 1000 mg/kg KG und Tag limitiert. Die gewählten Tetrahydrofuran-Dosen entsprachen ca. 235 bzw. 782 ml Tetrahydrofuran/m^3^. Die Dosen für C57BL/6-Mäuse betrugen 0, 1000 und 1500 mg/kg KG und Tag, die für C57BL/6-Doppelknockout-Mäuse 0 und 1500 mg/kg KG und Tag. Nach dem letzten Expositionstag wurden die Tiere getötet und untersucht. Es wurden keine Effekte auf Körpergewicht, relative und absolute Lebergewichte, Aspartat-Aminotransferase (AST), Alanin-Aminotransferase (ALT) und alkalische Phosphatase (ALP) festgestellt. Die histologische Untersuchung ergab keine Befunde in den Leberproben. Die hepatische mRNA wurde extrahiert und mit RT-PCR die mRNA der Gene *Cyp1a1*,* Cyp1a2*, *Cyp2b10*, *Cyp3a11*, *Cyp4a10* und *Acox1* gemessen. Zudem wurde der Gesamtgehalt an Cytochrom-P450 (CYP) sowie die Enzym-Aktivitäten von u. a. CYP1A1/2 mit dem Marker Ethoxyresorufin sowie von CYP2B10 und CYP3A11 mit den Markern Pentoxyresorufin und Benzyloxyresorufin untersucht. In den Proben der B6C3F1-Mäuse und C57BL6-Mäuse waren bei 1000 bzw. 1500 mg/kg KG und Tag die Zellproliferation (in Zone 3 der Leberproben) und die mRNA-Expression von *Cyp1a2* und *Cyp2b10* statistisch signifikant erhöht. Zudem waren der Gesamtgehalt an CYP-Enzymen und die Aktivitäten von CYP1A1/2 und CYP2B10 bei den Hochdosis-B6C3F1-Mäusen und den exponierten C57BL/6-Mäusen erhöht. In den Proben der Doppelknockout-Mäuse waren Cyp1a2-mRNA-Werte, CYP1A1/2-Aktivität und Zellproliferationen nicht verändert. Die Autoren vermuteten, dass die Enzyminduktionen bei B6C3F1- und C57BL/6-Mäusen auf einer Interaktion mit dem CAR/PXR zurückzuführen sind und stufen Tetrahydrofuran im Vergleich mit Phenobarbital als schwachen CAR/PXR-Aktivator ein (Choi et al. 2017). 

Gemäß dieser Studie ist eine Interaktion mit PPARα bzw. dem Arylhydrocarbonrezeptor (AhR) als Mechanismus der Tumorentstehung nicht zu vermuten, da weder erhöhte Werte von Cyp4a10-mRNA und Laurinsäure (Indikatoren für einen Mechanismus via PPARα) noch erhöhte Expression von Cyp1a1-mRNA (Indikator für einen Mechanismus via AhR) festgestellt wurden (Dekant 2019).

In der 13-Wochen-Studie des NTP wurde nur eine gering gesteigerte Zellproliferation in der Leber der B6C3F1-Maus bei 1800 ml/m^3^ festgestellt. Bei Studien mit 5-tägiger Exposition war diese deutlicher und reversibel, stieg aber nicht bei 20-tägiger Exposition an (Fenner-Crisp et al. 2011; Gamer et al. 2002; van Ravenzwaay et al. 2003).

Zudem hat der eingesetzte B6C3F1-Mausstamm eine hohe spontane Lebertumorrate. Eine Humanrelevanz von Lebertumoren, die nur nahe von toxischen Konzentration auftreten, ist als fraglich anzusehen (Laube et al. 2019). 

Es wird somit in der Gesamtschau der Daten angenommen, dass Tetrahydrofuran wie Phenobarbital über CAR/PXR wirkt. Tetrahydrofuran verursacht in Kurzzeit- bzw. Langzeitstudien eine gesteigerte Induktion von CAR/PXR-typischen CYP-Monooxygenasen und Zellproliferation in der Leber (Fenner-Crisp et al. 2011). Im Vergleich zu Phenobarbital ist Tetrahydrofuran nur ein schwacher CAR/PXR-Aktivator. Dies erklärt auch die lange Latenzzeit der Tumorentstehung in der NTP-Studie. Ein weiterer Beleg für diesen Mechanismus ist, dass die Lebereffekte bei weiblichen Mäusen auftraten, was konsistent mit den Ergebnissen einer Studie ist, in der weibliche Mäuse eine doppelt so hohe Expression von CAR in der Leber aufwiesen wie männliche (Choi et al. 2017). Entsprechend wurden bei männlichen Mäusen keine statistisch signifikant erhöhten Lebertumoren beobachtet. Allerdings war schon die Kontrollinzidenz der Neoplasien bei den männlichen Tieren sehr hoch, was den Nachweis einer geschlechtsspezifischen Wirkung erschwert.

Die Humanrelevanz der Tumorinduktion über den CAR/PXR-Mechanismus wird kontrovers diskutiert (Braeuning et al. 2014; Dekant 2019; Felter et al. 2018; Yamada et al. 2021) und kann nicht grundsätzlich ausgeschlossen werden.

### 2.5 Metabolomanalyse

Es wurden je fünf weibliche und männliche Wistar-Ratten (zehn Wochen alt) pro Gruppe inhalativ gegen 1800 oder 5000 ml/m^3^ sechs Stunden am Tag (entspricht 1422 bzw. 3950 mg Tetrahydrofuran/kg KG) und fünf Tage pro Woche, vier Wochen lang exponiert und eine Metabolomanalyse von 225 Metaboliten durchgeführt. Zusätzlich wurde jeweils fünf Wistar-Ratten pro Geschlecht und Dosis über eine Schlundsonde 1500 oder 2500 mg/kg KG verabreicht. Kontrollgruppen bestanden aus jeweils zehn männlichen und weiblichen Tieren. Das Blutplasma wurde am 6., 13. und 27. Tag bei inhalativer Exposition, sowie am 7., 14. und 28. Tag bei oraler Gabe massenspektrometrisch untersucht. Die oral exponierten weiblichen Tiere wurden aufgrund der Toxizität von Tetrahydrofuran nach 14 Tagen getötet und untersucht, die Tiere der anderen Gruppen nach der Expositionszeit von 28 Tagen. Etwa 80 bis 100 Metaboliten waren in ihrer Expression verändert. Bei den männlichen Tieren waren 34–55 % der Metaboliten, die nach Inhalation von 5000 ml/m^3^ statistisch signifikant verändert waren auch entsprechend bei oraler Gabe von 2500 mg/kg KG verändert. Bei den weiblichen Tieren betrug der Wert 19 %. Die häufigsten Veränderungen der Metabolitenprofile im Plasma traten bei Lipiden, Fettsäuren und Aminosäuren auf. Dieser Effekt war unabhängig vom Geschlecht und Expositionspfad. Die mengenmäßig häufigsten Metabolitenveränderungen im Vergleich zur inhalativen Exposition traten nach oraler Gabe auf, was mit der stärkeren Wirkung auf das Körpergewicht (Reduktion um 11 % nach oraler Gabe und keine Veränderung nach inhalativer Gabe) und der höheren Toxizität bei den weiblichen Tieren korreliert. Als Grund wurde die höhere Konzentration von Tetrahydrofuran in der Leber nach oraler Gabe angeführt. Die Leber wurde als Zielorgan identifiziert, narkotische und renale Wirkungen traten jedoch nicht auf. Die Veränderung in den Metabolit-Klassen der Lipide und Fettsäuren zwischen oraler Gabe und inhalativer Exposition sind laut der Autoren Anzeichen für eine Deregulation des Stoffwechsels in der Leber (Fabian et al. 2016).

## 3 Toxikokinetik und Metabolismus

### 3.1 Aufnahme, Verteilung, Ausscheidung

Tetrahydrofuran wird inhalativ und oral rasch aufgenommen und systemisch über das Blut verteilt. Die dermale Aufnahme aus der Gasphase bei Menschen hat nach Untersuchungen mit vier Probanden in Expositionskammern (150 ml Tetrahydrofuran/m^3^, vier Stunden lang, zwei Probanden mit und zwei ohne Atemmaske) einen Anteil an der inneren Tetrahydrofuran-Gesamtbelastung (gemessen in Ausatemluft, Blut und Urin) von 0,4 bis 5,9 %. Die Halbwertszeit für die Elimination aus dem Blut konnte mit bisher vorliegenden Daten nicht berechnet werden. Die Halbwertszeit von Tetrahydrofuran in der Exspirationsluft beträgt beim Menschen 30 Minuten (Greim 2003).

Bei 58 männlichen Beschäftigten (21–48 Jahre alt) mit einer durchschnittlichen Beschäftigungszeit von 7,2 Jahren, die gegen bis zu 200 ml Tetrahydrofuran/m^3^ (Beschichtungsprozess) oder 36 und 10 ml/m^3^ (Kalandrierung bzw. Verpackung) exponiert waren, betrugen die Korrelationskoeffizienten zwischen der Tetrahydrofurankonzentration in der Luft und denen in Urin, Blut und Ausatemluft 0,88; 0,68 bzw. 0,61 (Lewalter 1996). In der Studie wurde die dermale Exposition und mögliche dermale Aufnahme von Tetrahydrofuran nicht berücksichtigt. 

Folgende Untersuchungen sind neu hinzugekommen:

#### 3.1.1 Inhalation

##### 3.1.1.1 Mensch 

Vier Probanden resorbierten während einer 20-minütigen Exposition gegen 100 ml Tetrahydrofuran/m^3^ in Ruhe 60 % des Tetrahydrofurans. Laut der Autoren entsprach dieser Wert annähernd 80 % der Resorptionsquote im Fließgleichgewicht, das nach mehreren Stunden erreicht wird (US EPA 2012). 

In einer weiteren Studie wurden 1–20 männliche und weibliche Versuchspersonen gruppenweise (k. w. A.) über ein Mundstück sechs Minuten lang gegen 108–395 ml Tetrahydrofuran/m^3^ exponiert (Teramoto et al. 1988). Es wurde eine durchschnittliche Resorption von 64,8 % für männliche und von 72,7 % für weibliche Versuchspersonen berechnet, bei erhöhter Atmung 78,4 % bzw. 81,3 % (US EPA 2012). Es wurde nicht beschrieben, ob eine Abatmung über die Nase verhindert wurde.

Fünf männliche Versuchspersonen wurden in einer Expositionskammer drei Stunden lang gegen 56 ml Tetrahydrofuran/m^3^ exponiert. Während normaler Atemtätigkeit wurden 60 % und bei tiefen Atemzügen 73 % resorbiert. Ähnliche Aufnahmeanteile ergaben sich in einem identischen Experiment mit dreistündiger Exposition gegen 207 ml/m^3^ (Teramoto et al. 1988). Es wurde nicht beschrieben, ob eine Abatmung über die Nase verhindert wurde.

##### 3.1.1.2 Tier

Hierzu liegen keine neuen Daten vor.

#### 3.1.2 Orale Aufnahme

Männliche und weibliche F344-Ratten und B6C3F1-Mäuse erhielten eine einzelne Gabe von ^14^C-markiertem Tetrahydrofuran in wässriger Lösung via Schlundsonde. Die Dosen betrugen für männliche und weibliche Ratten 40,3 bzw. 45,9 und 428,7 bzw. 478,3 mg/kg KG. Für männliche und weibliche Mäuse betrugen die Dosen 44,3 bzw. 38,0 und 490,3 bzw. 495,9 mg/kg KG. Bis 168 Stunden nach Dosisgabe wurde in allen Gruppen die Radioaktivität in Urin, Faeces, abgeatmetem CO_2_, organischen, flüchtigen Anteilen, Geweben sowie die im Käfig verbliebene Radioaktivität bestimmt. Die Wiederfindung der Radioaktivität in den Hochdosisgruppen betrug für männliche und weibliche Ratten 33,0 bzw. 25,5 und für Mäuse 61,9 bzw. 43,3 %. In den Niedrigdosisgruppen ergaben sich für männliche und weibliche Ratten Werte von 67,5 bzw. 61,3 und für Mäuse 85,2 bzw. 108,5 %. Die geringe Wiederfindung bei den Experimenten mit hoher Dosierung ist laut den Autoren mit einer frühzeitigen Sättigung des Kollektors für das abgeatmete CO_2_ und unzureichender Leistung des Lösungsmittels für die abgeatmeten organischen Verbindungen zu erklären. Dieser Fehler wurde bei den Niedrigdosisgruppen der Mäuse behoben, sodass nur diese Daten für eine Auswertung als valide zu betrachten sind. Die höchsten Gewebekonzentrationen bei Ratten wurden in der Leber detektiert, gefolgt von Fettgewebe und Nebennieren. Bei Mäusen waren die Anteile in Fett und Nebennieren am höchsten. Bei beiden Spezies konnte Radioaktivität bereits zum frühesten Messzeitpunkt (15 Minuten nach Dosisgabe) im Plasma gemessen werden. Die maximalen Plasmakonzentrationen und die AUC („area under the curve“) bei Ratten waren nicht proportional zur Dosis. Dies ist einerseits mit der Sättigung der Resorption als auch mit möglichen dosisabhängigen Änderungen des First-Pass-Metabolismus zu erklären. Zudem legen die Daten eine schnellere Resorption von Tetrahydrofuran bei Mäusen als bei Ratten nahe und die AUC-Daten der männlichen Maus zeigen eine deutliche Überproportionalität der resorbierten Dosis zur applizierten Dosis: Die AUC war bei den männlichen und weiblichen Mäusen in der hohen Dosisgruppe im Vergleich zur jeweiligen Niedrigdosisgruppe 16- bzw. 12-mal so groß, die applizierten 11- bzw. 12-mal. Innerhalb von 168 Stunden wurden im Urin maximal ca. 5 % der applizierten Radioaktivität detektiert, in den Faeces 1,4 %. Die größten Mengen an Radioaktivität wurden als CO_2_ abgeatmet, bei Mäusen zudem ein relevanter Anteil der Radioaktivität als flüchtige organische Substanz. Tetrahydrofuran wird nach oraler Gabe an Mäuse nahezu vollständig resorbiert. Dies ist auch für Ratten anzunehmen. Als Halbwertszeit der Radioaktivität im Blutplasma wurde in der Hochdosisgruppe bei Ratten 53,5 ± 6,6 Stunden bestimmt. In der Niedrigdosisgruppe der Ratten 51,3 ± 2,8 Stunden (DuPont 1998).

#### 3.1.3 Dermale Aufnahme

In einer In-vitro-Studie, angelehnt an eine Prüfrichtlinie der US EPA zur dermalen Penetrationsbestimmung (40 CFR Parts 9 und 799, OFR und GPO 2022), wurde die Resorption von unverdünntem Tetrahydrofuran sowie 30- und 10%igen wässrigen Lösungen in Franz-Zellen (800 µl, 0,8 cm^2^, Rezeptor: physiologische Kochsalzlösung) an humaner Haut gemessen. Die Expositionszeiten betrugen 60 Minuten sowie 14 Stunden. Die 10- und 30%igen Lösungen hatten keinen signifikanten Einfluss auf die Barrierefunktion der Hautproben. Unverdünntes Tetrahydrofuran schädigte die Hautbarriere. Für die 10%ige Lösung und den Zeitraum von 1–6 Stunden betrug der Flux 1,360 ± 0,237 mg/cm^2^ und Stunde und der Permeabilitätskoeffizient 0,015 ± 0,003 cm/h. Die Werte im Zeitraum von 4–14 Stunden waren 1,006 ± 0,143 mg/cm^2^ bzw. 0,011 ± 0,002 cm/h. Basierend auf diesen Ergebnissen kann von einer schnellen Penetration von Tetrahydrofuran durch die humane Haut ausgegangen werden (ECHA 2022). Aus dem Flux nach 1–6 Stunden ergibt sich unter Standardbedingungen (eine Stunde, 2000 cm^2^) eine Aufnahme von 2720 mg.

#### 3.1.4 Fazit

Tetrahydrofuran wird von Mensch und Tier inhalativ, oral und dermal aufgenommen. In verschiedenen Studien an Probanden wurde eine inhalative Resorption in ähnlicher Höhe gezeigt. Die Resorptionsquote bei erhöhter Atemtätigkeit beträgt 73 %. Tetrahydrofuran wird mit dem Blut im ganzen Körper verteilt und vornehmlich als CO_2_ abgeatmet. Die Halbwertszeit von radioaktiv markiertem Tetrahydrofuran im Blutplasma beträgt bei Ratten nach oraler Gabe 51,3–53,5 Stunden. Die Halbwertszeit von Tetrahydrofuran im Blut beim Menschen ist nicht genau bekannt.

### 3.2 Metabolismus

Tetrahydrofuran wird von Ratten nach inhalativer Exposition von induzierbaren Enzymen metabolisiert (Greim 2003). Konkretere Angaben zum Metabolismus konnten bisher nicht getroffen werden. Folgende Studien sind neu hinzugekommen:

#### 3.2.1 In vitro

Lebermikrosomen von Ratten, Mäusen und Menschen wurden in Anwesenheit von NADPH bis zu 55 Minuten mit 70 µM (5047,7 µg/l) Tetrahydrofuran inkubiert. Als einziger Metabolit wurde Gammahydroxybuttersäure identifiziert. Diese kann über den Citratzyklus weiter zu CO_2_ metabolisiert werden. Die Halbwertszeit von Tetrahydrofuran betrug 40,1; 28 und 9 Minuten mit Ratten-, Menschen- bzw. Mäuse-Mikrosomen. Es wurden bei Mäusen keine geschlechtsspezifischen Unterschiede in der Umsatzgeschwindigkeit festgestellt. Die intrinsische, mit zwei Methoden bestimmte Clearance betrug mit Mikrosomen von Maus, Ratte oder Mensch 160,5; 30,6 bzw. 27,31 ml/min/kg KG und 205,4; 30,6 bzw. 28,6 ml/min/kg KG (ECHA 2022). Der Vergleich des hepatischen Blutflusses mit der intrinsischen Clearance zeigt einen First-Pass-Effekt bei Maus und Mensch. Zwar wurden die für den Metabolismus verantwortlichen Enzyme nicht identifiziert, aber da Mikrosomen in Anwesenheit von NADPH verwendet wurden, sind vermutlich ein oder mehrere CYP-Enzyme beteiligt (US EPA 2012). 

#### 3.2.2 Mensch 

Im Rahmen eines Fallberichts einer Tetrahydrofuranvergiftung wurde in Blut und Serum einer weiblichen Person (k. w. A.) Gammahydroxybuttersäure nachgewiesen (Cartigny et al. 2001).

#### 3.2.3 Tier

Nach oraler Gabe wurde ^14^C-Tetrahydrofuran von B6C3F1-Mäusen und F344-Ratten hauptsächlich zu CO_2_ metabolisiert und abgeatmet. Mäuse atmeten auch einen relevanten Anteil als flüchtige organische Substanz ab (Abschnitt 3.1), die nicht weiter identifiziert wurde. Es ist möglich, dass es sich hierbei um die Ausgangssubstanz handelte. Da der Abbau von Tetrahydrofuran zu CO_2_ in der Niedrigdosisgruppe ausgeprägter war, lässt sich eine Sättigung des Metabolismus bei höheren Dosen vermuten. Aufgrund der beschriebenen Sättigung der CO_2_-Falle in den Versuchen, ist ein speziesabhängiger Unterschied im Metabolismus von Tetrahydrofuran bei Ratten und Mäusen zu vermuten, jedoch ist dieser nicht abschließend belegt (DuPont 1998). 

**Abb.1:**
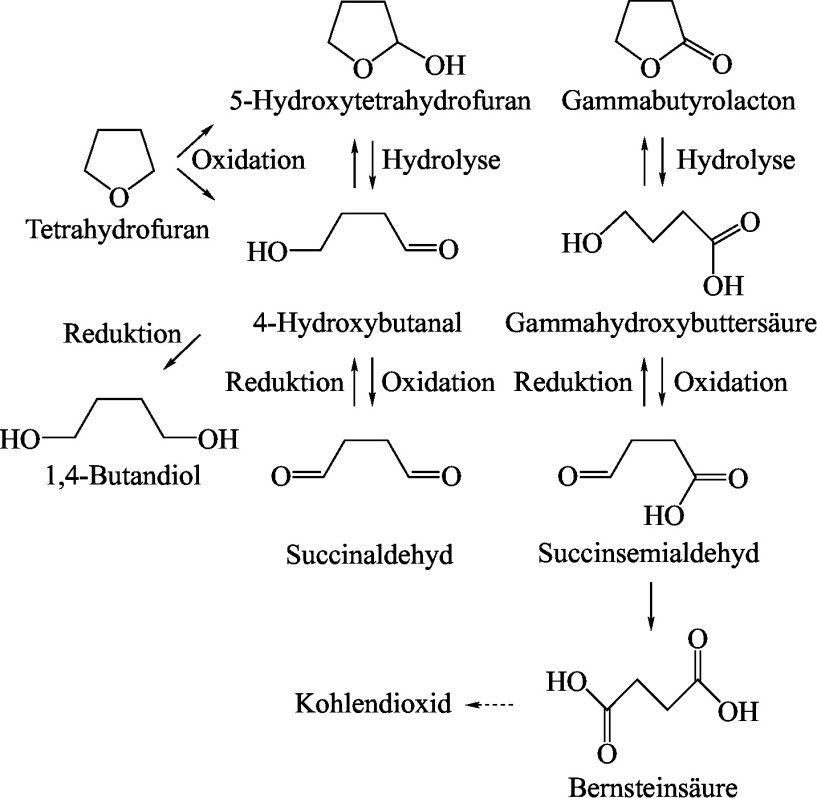
Metabolismusschema von Tetrahydrofuran nach US EPA (2012)

Weitere Untersuchungen mit zu Tetrahydrofuran strukturverwandten Substanzen lassen dessen Umwandlung in Gammahydroxybuttersäure über verschiedene Reaktionen im Metabolismus vermuten. Hierzu zählen die α-Oxidation von Tetrahydrofuran durch CYP zu 5-Hydroxytetrahydrofuran, welches dann rasch zu Gammabutyrolacton und Gammahydroxybuttersäure umgewandelt wird. Des Weiteren ist eine Oxidation mittels zytosolischer Enzyme (Dehydrogenasen) zu 4-Hydroxybutanal möglich, welches zu Gammabutyrolacton und Gammahydroxybuttersäure oxidiert oder zu 1,4-Butandiol reduziert wird. Zudem ist eine direkte Oxidation zu Succinaldehyd mittels CYP möglich, welches entweder zu 4-Hydroxybutanal reduziert und zu Gammabutyrolacton oxidiert wird oder direkt zu Gammabutyrolacton und Gammahydroxybuttersäure oxidiert wird. Es ist zudem bekannt, dass Gammabutyrolacton in wässrigem Medium zu Gammahydroxybuttersäure hydrolysiert. Die Hydrolyse von Gammabutyrolacton zur hydroxylierten Säure und die umgekehrte Reaktion von Gammahydroxybuttersäure zum Lacton durch humane Paraoxonase 1 wurde nachgewiesen. Gammahydroxybuttersäure kann mittels der zytosolischen NADP^+^-abhängigen Gammahydroxybuttersäure-Dehydrogenase zu Succinsemialdehyd umgewandelt werden, Succinsemialdehyd wiederum zu Bernsteinsäure. Diese wird im Citratzyklus letztlich zu CO_2_ umgesetzt. GABA kann aus Gammahydroxybuttersäure entstehen, wurde aber als Metabolit von Tetrahydrofuran nicht nachgewiesen (US EPA 2012). Das auf diesen Untersuchungen basierende Metabolismusschema von Tetrahydrofuran ist in Abbildung 1 dargestellt.

#### 3.2.4 Fazit

Tetrahydrofuran wird oxidativ durch CYP metabolisiert. Eine zusätzliche Oxidation erfolgt mittels zytosolischer Dehydrogenasen. Tetrahydrofuran wird zu den Zwischenprodukten 5-Hydroxytetrahydrofuran und 4-Hydroxybutanal metabolisiert. Basierend auf den vorliegenden Untersuchungen wird postuliert, dass diese über weitere Oxidation zu Gammabutyrolacton, Gammahydroxybuttersäure und Succinaldehyd umgewandelt werden. Die daraus entstehende Bernsteinsäure gelangt in den Citratzyklus wo sie zu CO_2_ umgesetzt wird, dem Hauptmetabolit von Tetrahydrofuran. Die Umwandlung von Gammahydroxybuttersäure zu GABA, einem bekannten neurotoxischen Stoff, ist anzunehmen. 

## 4 Erfahrungen beim Menschen

### 4.1 Einmalige Exposition

Arbeiter, die einer unbekannten Tetrahydrofurankonzentration ausgesetzt waren, berichteten über Übelkeit, Kopfschmerzen, Sehstörungen, Brustschmerzen, Luftnot und Husten und es wurde ein Anstieg von Leberenzymaktivitäten festgestellt. Andere klinische oder biologische Effekte wurden nicht beobachtet. Die Symptomatik und die Leberfunktionsstörung bildeten sich innerhalb von zwei Tagen wieder zurück (Lewalter 1996).

### 4.2 Wiederholte Exposition

Exposition gegen Tetrahydrofuran oder Mischungen, die Tetrahydrofuran und andere potentiell hepatotoxische Lösungsmittel enthalten, können zu erhöhten Aktivitäten der Leberenzyme im Serum führen (Lewalter 1996). Eine erhöhte Serum-AST-Aktivität wurde bei Arbeitern in einem PVC-verarbeitenden Betrieb gefunden, die gegenüber maximal 1000 ml/m^3^ exponiert waren (Greim 2003). Folgende Studien sind neu hinzugekommen:

Während der Exposition gegen Tetrahydrofuran und andere Lösungsmittel, wie Aceton und Cyclohexan, entwickelte ein Beschäftigter (41 Jahre alt, Raucher) einen leicht erhöhten Nasenausfluss und er berichtete über einen unangenehmen Geruch bzw. Geruchsverlust über einen Zeitraum von zehn Wochen. Sechs Wochen nach Expositionsende kehrte die Geruchswahrnehmung zurück, war aber nach sieben Monaten immer noch reduziert (Emmett 1976).

In einem Werk zur Schlauchherstellung wurden Interviews mit 35–40 Beschäftigten zu ihrem Gesundheitszustand durchgeführt. Die Beschäftigten berichteten Augen- und Atemwegsreizungen, Kopfschmerzen, Benommenheit und Müdigkeit. Alveolengängiger und einatembarer Staub, alveolengängiges Siliciumdioxid, Metalle, Tetrahydrofuran, Nitrosamine und weitere organische Lösungsmittel (Aceton, Toluol, Methylethylketon und 1,1,1-Trichlorethan) wurden in der Luft gemessen. In fünf Proben ergaben sich Konzentrationen von 20–83 ml Tetrahydrofuran/m^3^. Da es am Probenahme-Gerät zu Durchbrüchen kam, waren die Konzentrationen vermutlich noch höher (US EPA 2012).

Ein weiterer Fallbericht liegt zu einem 28-jährigen Beschäftigten vor, der neun Jahre lang mit Rohrzement arbeitete. Hierbei traten mehrfach 15-minütige Expositionen gegen 389–757 ml Tetrahydrofuran/m^3^ sowie gegen Cyclohexan und Methylketon in unbekannter Höhe auf. Der Beschäftigte wies eine Hämaturie auf. Eine Biopsie der Niere zeigte eine proliferative Glomerulonephritis mit Immunglobulin-A-Ablagerungen, Adhäsion von Kapillaren an die Bowman-Kapsel sowie Fibrin in den glomerulären Ablagerungen des Mesangiums (Albrecht et al. 1987). Ein kausaler Zusammenhang mit der Exposition gegen Tetrahydrofuran ist unklar und der Einfluss der Exposition gegen die oben genannten weiteren Chemikalien ist nicht aufgeklärt worden.

**Fazit**: Die neu hinzugekommenen Studien zur einmaligen und wiederholten Exposition sind aufgrund fehlender Angaben zur Expositionshöhe oder wegen Ko-Exposition nicht für eine Bewertung von Tetrahydrofuran geeignet.

### 4.3 Wirkung auf Haut und Schleimhäute

Akute Reizwirkungen auf den Atemtrakt wurden bei einer dreistündigen Exposition gegen 50 oder 200 ml Tetrahydrofuran/m^3^ in Probandenstudien zur Toxikokinetik nicht berichtet (Abschnitt 3.1.1.1). Die Geruchsschwelle für Tetrahydrofuran wurde mit 30 ml/m^3^ angegeben (Greim 2003). Folgende Informationen sind neu hinzugekommen:

In einer Studie aus dem Jahr 1954 wurde sechs Probanden Tetrahydrofuran auf die Haut aufgetragen (k. w. A.) und eine Reizwirkung festgestellt. Diese war schwerwiegender, wenn die Substanz nach Applikation verdunsten konnte. Die Autoren führten die Reizung auf Verunreinigungen in der Lösung zurück, die nach Verdunsten auf der Haut verblieben (US EPA 2012). Aufgrund der fehlenden Angaben zu den Versuchspersonen und der Art und Dauer der Applikation ist die Studie nicht für die Bewertung geeignet. 

In Sicherheitsdatenblättern wird auf ein entfettendes Potential von Tetrahydrofuran nach Hautkontakt hingewiesen (Merck KGaA 2023).

### 4.4 Allergene Wirkung

Eine allergene Wirkung konnte bislang nicht zweifelsfrei belegt werden (Greim 2003).

In einer sekundär zitierten Untersuchung aus dem Jahr 1938 konnte an 196 Freiwilligen keine Kontaktdermatitis oder Sensibilisierung durch Tetrahydrofuran beobachtet werden (US EPA 2012). Es fehlen jegliche weitere Informationen zu der Versuchsdurchführung und den eingesetzten Konzentrationen, weshalb die Untersuchung nicht zur Bewertung von Tetrahydrofuran herangezogen wird.

### 4.5 Reproduktionstoxizität

Hierzu liegen keine Daten vor.

### 4.6 Genotoxizität

Lymphozytenkulturen von sieben Beschäftigten (25–49 Jahre alt, Expositionszeitraum 2–28 Jahre) aus chemischen Laboratorien, exponiert gegen Tetrahydrofuran als Lösungsmittel, wiesen eine statistisch signifikant erhöhte Anzahl an Chromosomenbrüchen im Vergleich zu einer Kontrollgruppe von 42 Erwachsenen (18–63 Jahre alt, k. w. A.) auf. Die Anzahl der Chromosomenbrüche (ohne Gaps) der exponierten Personen betrug durchschnittlich 9,4 pro 100 Zellen, die der Kontrollgruppe 5,6 pro 100 Zellen. Die Häufigkeit abnormer Zellen betrug bei den Exponierten 7,7 % im Vergleich zu 4,8 % bei den Kontrollpersonen. Vier der sieben exponierten Personen waren auch gegen Benzol exponiert (k. w. A.; Funes-Cravioto et al. 1977). Da im Bericht relevante Daten zu den Expositionsbedingungen fehlen und eine Exposition gegen andere Substanzen mit klastogener Wirkung (Benzol) beschrieben ist, wird diese Studie für die Bewertung von Tetrahydrofuran nicht herangezogen.

### 4.7 Kanzerogenität

Bisher lagen keine epidemiologischen Daten vor (Greim 2003). 

ICARE ist eine große, bevölkerungsbezogene Fall-Kontroll-Studie, die in Frankreich durchgeführt wurde. Erfasst wurde das lebenslange Berufsleben, Tabakrauchen und Alkoholkonsum. Untersucht wurde eine mögliche Assoziation einer Exposition gegen Erdöl-basierte und oxygenierte Lösungsmittel mit der Inzidenz von Krebs des Hypopharynx und des Kehlkopfes. Das untersuchte Kollektiv bestand aus 383 Fällen von Krebs des Hypopharynx, 454 Fällen von Kehlkopfkrebs und 2780 Kontrollen. Es wurde eine Job-Expositions-Matrix (JEM) verwendet, um eine Exposition gegen Erdöl-basierte Lösungsmittel wie Benzol, Benzin, Testbenzin, Diesel und Kerosin und oxygenierte Lösungsmittel wie Alkohole, Ketone und Ester, Ethylenglykol, Diethylether und Tetrahydrofuran zu erfassen. Die Fall-Kontroll-Studie wurde im Zeitraum von 2001 bis 2007 in Frankreich in zehn geographischen Regionen mit Krebsregister durchgeführt. Die Patienten waren im Alter zwischen 18 und 75 Jahren. Die Kontrollpersonen waren aus den gleichen geographischen Regionen und wurden nach Alter und Geschlecht angepasst. Die Daten wurden mit Hilfe eines Fragebogens in einem persönlichen Interview erhoben, dabei wurden auch der Alkoholkonsum und der sozioökonomische Status des Kollektivs miterfasst. Adjustiert wurde nach Alter, Wohnsitz, Raucherstatus, Tabakkonsum, Alkoholkonsum und der kumulativen Asbest-Exposition. Die Exposition gegen Tetrahydrofuran war nicht statistisch signifikant mit erhöhten Risiken für Krebs des Hypopharynx (OR (Odds Ratio): 1,67; 95-%-KI (Konfidenzintervall): 0,87–3,21) oder des Kehlkopfes (OR: 1,39; 95-%-KI: 0,73–2,63) assoziiert. In Abhängigkeit von der kumulativen Exposition nahmen die OR für Krebs des Hypopharynx nicht statistisch signifikant zu (höchste Expositionsgruppe, n = 6, OR: 2,63; 95-%-KI: 0,55–12,65; p (Trend): 0,07). Keiner der Fälle war nur gegen Tetrahydrofuran exponiert (Barul et al. 2018). Die Studie ist als valide einzustufen. Die Autoren diskutieren eine mögliche Fehlklassifikation bezüglich der Exposition, da die JEM die unterschiedliche Aufgabenverteilung innerhalb derselben Berufsbezeichnung nicht berücksichtigt. Andererseits weisen JEM die Exposition auf reproduzierbare und automatische Weise zu, unabhängig vom Fall- oder Kontrollstatus; folglich ist eine Fehlklassifizierung der Exposition wahrscheinlich nicht-differentiell. Mögliche Klassifikationsfehler bezüglich des Krankheits- und des Expositionsstatus sind somit unabhängig voneinander. Die Expositionserhebung wird daher wahrscheinlich zu einer Unterschätzung des Risikos führen. Ein Recall-Bias ist nicht auszuschließen, mit der Verwendung eines standardisierten Fragebogens wurde dieser jedoch versucht zu minimieren. Es ist zu berücksichtigen, dass eine Vielzahl an Assoziationen untersucht worden sind, sodass auch Zufallsergebnisse zu erwarten sind. Da keiner der Fälle nur gegen Tetrahydrofuran exponiert war, ist eine Aussage zur kanzerogenen Wirkung nicht möglich. 

Im Rahmen der ICARE-Studie wurde eine weitere Analyse einer möglichen Assoziation zwischen einer Exposition gegen Tetrahydrofuran und Krebs der Mundhöhle sowie des Mundrachens durchgeführt. Einbezogen wurden 350 Fälle von Plattenepithelkarzinomen der Mundhöhle und 543 Krebsfälle des Mundrachens (Oropharynx) sowie die 2780 Kontrollpersonen (siehe Barul et al. 2018). Für Patienten, die jemals gegen Tetrahydrofuran exponiert waren, wird ein nicht statistisch signifikant erhöhtes OR von 1,87 (95-%-KI: 0,97–3,61) für Krebs der Mundhöhle angegeben, adjustiert für Alkoholkonsum und Raucherstatus. Nach Adjustierung für Expositionen gegen andere Lösungsmittel blieb das erhöhte Risiko bestehen. Es wurde keine Expositionsabhängigkeit beobachtet (p (Trend): 0,52). Für das Auftreten von Oropharynx-Krebsfällen ergaben sich keine erhöhten OR (Barul et al. 2019). Die Autoren geben an, dass sie das Ausmaß der Fehlklassifikation bei dieser Analyse nicht abschätzen können. Angaben zur Mundhygiene und Ernährung liegen nicht vor. 

**Fazit**: Die populationsbasierten Fall-Kontroll-Studien zur Assoziation zwischen Tetrahydrofuran und Krebs des Hypopharynx, Kehlkopfs und der Mundhöhle ergaben nicht statistisch signifikant und nur geringfügig erhöhte OR. Eine Expositionsabhängigkeit wurde nicht beobachtet. Für den Oropharynx war das Krebsrisiko nicht erhöht. Da keiner der Fälle allein gegen Tetrahydrofuran exponiert war, sowie Fehlklassifikationen nicht auszuschließen sind, können die Daten zur Bewertung der kanzerogenen Wirkung von Tetrahydrofuran nicht herangezogen werden. 

## 5 Tierexperimentelle Befunde und In-vitro-Untersuchungen

### 5.1 Akute Toxizität

Die LC_50_ für Ratten bei 4-stündiger Exposition lag bei etwa 18 000 ml Tetrahydrofuran/m^3^, die orale LD_50_ bei 2,3–3,6 ml/kg KG (Greim 2003). Die Untersuchungen zu Zilienschlagfrequenzen und Reizwirkung am Atemtrakt sind für die Reevaluierung des MAK-Werts erneut geprüft worden:

Bei Kaninchen (k. w. A.) wurde unmittelbar nach 4-stündiger Exposition gegen 0, 100, 250, 1000, 6000 oder 12 000 ml Tetrahydrofuran/m^3^ ab 100 ml/m^3^ eine konzentrationsabhängig reduzierte Schlagfrequenz der Zilien in Trachea- und Nasenschleimhaut festgestellt. Die Zilienschlagfrequenz erreichte 1–3 Stunden nach Exposition wieder Normalwerte (ca. 800 Schläge pro Minute), in den beiden obersten Konzentrationsgruppen jedoch nicht. In der elektronenmikroskopischen Untersuchung waren ab 250 ml/m^3^ die Epithelzellen sporadisch vakuolisiert und Zilien miteinander verbunden. Ab 1000 ml/m^3^ wurden verstärktes Verkleben der Zilien und aufgeblähte, nicht ziliierte Zellen festgestellt. Die LOAEC betrug 100 ml Tetrahydrofuran/m^3^. Eine NOAEC konnte nicht ermittelt werden (Ikeoka et al. 1984; Ohashi et al. 1982 a, 1983). 

Seit Erscheinen der Begründung (Greim 2003) sind die folgenden Studien zur akuten Wirkung von Tetrahydrofuran hinzugekommen:

#### 5.1.1 Inhalative Aufnahme

Je ein bis zwei Katzen (k. w. A.) wurden drei bis acht Stunden gegen 3340, 16 700, 33 400, 66 800 oder 133 600 ml Tetrahydrofuran/m^3^ ganzkörperexponiert. Angaben zur Reinheit der Testsubstanz liegen nicht vor. Keines der Tiere starb bei 3340 oder 16 700 ml/m^3^, wohingegen alle Tiere bei 66 800 bzw. 133 600 ml/m^3^ starben. Zwölf Tage nach der Exposition wurden in allen Konzentrationsgruppen bei allen Tieren folgende Befunde verzeichnet: Speichel- und Tränenfluss, Nasenausfluss, Schwindel und Schläfrigkeit, vermehrtes Blinzeln. Bei höheren Konzentrationen oder längerer Exposition (k. w. A.) zeigte sich eine vermehrte Bauch- und Seitenlage, Narkose, flacher Muskeltonus und unregelmäßige Atmung (DuPont 2010). Eine LC_50_ wurde nicht angegeben. 

Je drei männliche und weibliche Sprague-Dawley-Ratten (k. w. A.) wurden 30 Minuten bzw. eine Stunde lang gegen 125 317 oder 101 085 ml Tetrahydrofuran/m^3^ ganzkörperexponiert. Zusätzlich wurden jeweils sechs weibliche und männliche Tiere zehn Minuten gegen 110 914 ml/m^3^ exponiert. Angaben zur Reinheit der Testsubstanz liegen nicht vor. Es traten Narkose, Atemwegsreizungen, sedative Wirkungen und Mortalität auf. Die histopathologische Untersuchung ergab eine akute Erweiterung des rechten Herzventrikels, kongestives Herzversagen und aufgeblähte Lungen. Die Mortalität betrug 0/12, 3/6 und 6/6 nach 10-, 30- bzw. 60-minütiger Exposition. Die LC_50_ lag bei 125 250 ml Tetrahydrofuran/m^3^ nach 30 Minuten Expositionszeit (DuPont 2010).

Je sechs männliche CD-Ratten (ca. acht Wochen alt) wurden einmalig gegen 3010, 4900, 5380, 5920, 6590, 6690, 6830, 13 200 oder 20 500 ml Tetrahydrofuran/m^3^ sechs Stunden lang ganzkörperexponiert. Zusätzlich wurden sechs weibliche CD-Ratten gegen 5700 ml/m^3^ sechs Stunden lang ganzkörperexponiert und anschließend 14 Tage lang nachbeobachtet. Angaben zur Reinheit der Testsubstanz liegen nicht vor. Keines der Tiere starb. Es wurden gesteigerte Atemfrequenz, verringerte oder ausbleibende Reaktion auf akustische Reize und narkotische Wirkungen festgestellt. Die nicht narkotische Konzentration betrug für männliche und weibliche Ratten 5380 bzw. 5700 ml Tetrahydrofuran/m^3^. Die LC_50_ war > 20 500 ml/m^3^ (ECHA 2022).

Je zwölf männliche und weibliche CD-Ratten (6–8 Wochen alt) pro Gruppe wurden einmalig sechs Stunden lang gegen 0, 500, 2500 oder 5000 ml Tetrahydrofuran/m^3^ ganzkörperexponiert. Die Reinheit der Substanz war > 99 %. Das Verhalten der Tiere (motorische Aktivität und Verhaltenstests („functional observational battery“)) wurde vor, am Ende und am ersten, siebten und 14. Tag nach der Exposition untersucht. Keines der Tiere starb. Es wurde eine konzentrationsabhängige sedative Wirkung berichtet. Nach zwei Stunden Exposition gegen 2500 oder 5000 ml/m^3^ trat eine verringerte Reaktion auf einen akustischen Stimulus bei 4/24 bzw. 8/24 Tieren auf (das Geschlecht der Tiere wurde bei diesen Ergebnissen nicht separat aufgeführt). Nach sechs Stunden waren alle Tiere der 5000-ml/m^3^-Gruppe lethargisch und es traten Gang-Anomalien, Veränderungen des Stellreflexes und Lidschlusses auf. Die Verhaltenseffekte waren vollständig reversibel (einige Stunden nach Exposition gegen 2500 ml/m^3^, 19 Stunden nach Exposition gegen 5000 ml/m^3^). An den ersten beiden Tagen waren die Futteraufnahme und das Körpergewicht der weiblichen Ratten nach Exposition gegen die höchste Konzentration statistisch signifikant reduziert (Malley et al. 2001).

#### 5.1.2 Orale Aufnahme

Je drei männliche und weibliche Wistar-Ratten (k. A. zum Alter der Tiere) pro Dosisgruppe erhielten einmalig mit der Schlundsonde 500, 1260, 2000, 2520 oder 4000 mg Tetrahydrofuran/kg KG. Angaben zur Reinheit der Testsubstanz liegen nicht vor. Die Tiere wurden 60 Minuten, drei, sechs und 24 Stunden nach Dosisgabe untersucht. Eine weitere Befundung der Tiere erfolgte jeweils alle 24 Stunden, 14 Tage lang. Bei der höchsten Dosis starben alle Tiere bereits vor Ende der Versuchslaufzeit. Histopathologische Untersuchungen stellten u. a. fibrotisches Gewebe um Herz und Lungen, Rötungen der Darmschleimhaut und Verdickungen der Magenwand bei 500 mg/kg KG fest. Die LD_50_ betrug 1650 mg/kg KG (GAF Corporation 1978). 

Eine Studie zur akuten Toxizität aus dem Jahr 1971 untersuchte Effekte von Tetrahydrofuran (Reinheit: analytischer Grad) an je sechs bis zwölf weiblichen und männlichen Sprague-Dawley-Ratten (1–2 Tage alt), jungen Ratten (14 Tage alt), jungen ausgewachsenen Ratten (k. A. zum Alter) sowie alten Ratten (k. A. zum Alter) nach oraler Gabe. Die LD_50_-Werte in den vier Altersgruppen betrugen < 1; 2,3; 3,6 bzw. 3,2 ml/kg KG (ECHA 2022).

Hunde (k. w. A.) erhielten einmalig ca. 0,3 ml Tetrahydrofuran/kg KG (zwei Tiere)oder 0,45 ml/kg KG (ein Tier) in einer 15%igen wässrigen Lösung. Blutproben wurden am Expositionstag entnommen. Keines der Tiere starb aufgrund der Exposition. Alle Tiere wiesen eine leichte Narkose auf, taumelten und erbrachen sich. Die Tiere wurden sieben Tage lang nachbeobachtet. Es wurde eine Reizung der Magenschleimhaut beobachtet. Bei der höchsten Dosis wurde ein erhöhter Gesamtproteingehalt im Blut festgestellt (DuPont 2010).

Je ein bis zwei Kaninchen (k. w. A.) erhielten einmalig mit der Schlundsonde 10–25%ige Tetrahydrofuran-Lösungen in Dosen von 1000, 2000, 2500 oder 3000 mg/kg KG. Die Tiere wurden 38 Tage lang nachbeobachtet. Alle Tiere, die 2500 oder 3000 mg/kg KG erhielten, starben. Bei 1000 mg/kg KG waren die Tiere ab sechs Stunden nach Gabe geschwächt und torkelten. Bei 2000 mg/kg KG wurden reduzierte Futteraufnahmen, Körpergewichtsverluste, reduzierte körperliche Aktivität sowie Effekte an der Leber und den Nieren (k. w. A.) festgestellt (DuPont 2010). Eine LD_50_ wurde nicht angegeben.

#### 5.1.3 Dermale Aufnahme

In einer Studie gemäß OECD-Prüfrichtlinie 402 wurden je fünf männliche und weibliche Wistar-Ratten 24 Stunden lang gegen 2000 mg Tetrahydrofuran/kg KG semi-okklusiv exponiert. Die Reinheit der Testsubstanz war > 99 %. Es traten weder Mortalität, Anzeichen von toxischer Wirkung noch Reizeffekte an der Haut (siehe Abschnitt 5.3.1) auf. Die LD_50_ war größer als 2000 mg/kg KG (ECHA 2022).

#### 5.1.4 Fazit

Bei Kaninchen (k. w. A.) waren unmittelbar nach einer inhalativen Exposition ab 100 ml Tetrahydrofuran/m^3^ die Schlagfrequenzen der Zilien in Trachea- und Nasenschleimhaut reduziert. Die letale Wirkung der Substanz steht im Zusammenhang mit ihrer narkotischen Wirkung.

### 5.2 Subakute, subchronische und chronische Toxizität

#### 5.2.1 Inhalative Aufnahme

Die Wirkungen von Tetrahydrofuran nach wiederholter inhalativer Verabreichung sind in Tabelle 1 dargestellt. Ergebnisse aus mechanistischen, subakuten Inhalationsstudien von Gamer (2002) und van Ravenzwaay (2003) sind in der Begründung (Greim 2003) beschrieben, ebenso wie die Studien aus Japan (Katahira 1982; Ohashi et al. 1982 a, b). Die niedrigste systemische NOAEC ist 500 ml/m^3^ aus einer 13-Wochen-Studie an Ratten, bei denen bei 1500 ml/m^3^ narkotische Wirkungen auftraten (Malley et al. 2001). Untersuchungen zu Zilienschlagfrequenzen und Reizeffekten (Katahira 1982; Ohashi et al. 1982 a, b) sowie die nicht-neoplastischen Befunde aus einer 13-Wochen-Studie (Chhabra et al. 1990; Vorstudie zu den Langzeituntersuchungen zur kanzerogenen Wirkung (NTP 1998)) sind im Rahmen der Neubewertung des Wirkmechanismus sowie des MAK-Werts erneut aufgeführt:

**Tab.1:** Toxizität von Tetrahydrofuran nach wiederholter inhalativer Exposition

Spezies, Stamm, Anzahl pro Gruppe	Exposition^a)^	Befunde	Literatur
Ratte, Sprague Dawley, k. w. A.	7 d, 3 Wo, 0, 100, 5000 ml/m^3^, 4 h/d, 5 d/Wo, Reinheit: k. A.	**ab 100 ml/m^3^**: **LOAEC** Tracheaschleimhaut: sekretorische Granula verändert, Schleimabsonderungen u. interzellulärer Abstand ↑, Nasenschleimhaut: Granula ↑, Vakuolen im Zytoplasma, Verlust von Zilien, Rupturen der Zellmembranen der Zilien-tragenden Zellen; **bei 100 ml/m^3^**: Tracheaschleimhaut: Zilienschlagfrequenz nach 7 d und 3 Wo ↓ (5 bzw. 11 %), Nasenschleimhaut: Zilienschlagfrequenz nach 7 d und 3 Wo ↓ (11 bzw. 39 %); **bei 5000 ml/m^3^**: Tracheaschleimhaut: Zilienschlagfrequenz nach 7 d und 3 Wo ↓ (18 bzw. 24 %), Nasenschleimhaut: Zilienschlagfrequenz nach 7 d und 3 Wo ↓ (28 bzw. ca. 10 %)	Ohashi et al. 1982 b
Ratte, Sprague Dawley, 10 ♂	12 Wo, 0, 200, 1000 ml/m^3^, 4 h/d, 5 d/Wo, Reinheit: k. A.	**ab 200 ml/m^3^**: **LOAEC ** ähnliche morphologische Befunde wie in Ohashi et al. (1982 b), Tracheaschleimhaut: vermehrt Schleim an den Zilien, Schwellung der Zilienmembran	Ohashi et al. 1982 a
Ratte, Sprague Dawley, 11–12 ♂	12 Wo, 0, 100, 200, 1000, 5000 ml/m^3^, 4 h/d, 5 d/Wo, Reinheit: k. A.	**ab 100 ml/m^3^**: ** LOAEC ** Nasenschleimhaut: leichte Irritationen, Tracheaschleimhaut: vermehrt Granula sowie Vakuolen im Zytoplasma und Rupturen der Zellmembranen der Zilien-tragenden Zellen; **ab 1000 ml/m^3^**: AST- und Cholinesterase-Aktivitäten im Serum ↑; **5000 ml/m^3^**: deutliche lokale Reizwirkungen (Speichelfluss, Absonderungen und Blutungen aus der Nase, Ödeme und Trübung der Hornhaut), Bilirubin ↑, KG ↓, rel. Milzgewicht ↓	Katahira 1982
Ratte, Wistar, 5 ♂ (Kontrolle), 25 ♂ exponiert	12 Wo, 0, 3000 ml/m^3^, 1 h/d, 5 d/Wo, Reinheit: k. A.	**bei 3000 ml/m^3^**:** LOAEC** KG ↓, Tränenfluss, Speichelfluss, blutiger Nasenausfluss, Bronchien: papilläre Hyperplasien und Degenerationen im Epithel, Lunge: fokale Entzündungen, Degenerationen, Niere: Proteinzylinder, hyaline Tröpfchen, Degeneration	Kawata und Ito 1984
Ratte, CD, 18 ♂, 18 ♀ (Kontrolle und 3000 ml/m^3^); 12 ♂, 12 ♀ (500 und 1500 ml/m^3^)	13 Wo, 0, 500, 1500, 3000 ml/m^3^, 6 h/d, 5 d/Wo, Reinheit: > 99 %, Peroxidgehalt: < 50 mg/l	**500 ml/m^3^**: **NOAEC** **ab 1500 ml/m^3^**: ♂, ♀: verringerte Reaktion auf akustischen Stimulus aufgrund narkotischer Wirkung (reversibel), Nasen- und Augenausfluss, rötliche und braune Flecken nahe Nase und Auge	Malley et al. 2001
Ratte, F344, 10 ♂, 10 ♀	13 Wo, 0, 66, 200, 600, 1800, 5000 ml/m^3^, 6 h/d, 5 d/Wo, Reinheit: 99 %, Peroxidgehalt: < 1,5 mg/l	**< 5000 ml/m^3^ (k. w. A.)**: Nasen- und Augenausfluss; **5000 ml/m^3^**:♂, ♀: Ataxien,rel. und abs. Gewicht Milz und Thymusdrüse ↓, Erythrozytenanzahl ↑, Hämoglobin ↑, mittleres korpuskuläres Volumen ↑, ♂: mittleres korpuskuläres Hämoglobin ↑, absolute Neutrophilenzahl ↑, Vormagen: Akanthose (♂: 5/10, ♀: 8/10), Entzündung (♂: 2/10, ♀: 4/10), ♂: rel. Gewicht Leber, Niere, Lunge ↑, ♀: rel. und abs. Lebergewicht, Blutplättchen ↑, Gallensäure im Serum ↑, Blut-Harnstoff-Stickstoff- und Kreatiningehalt ↓	Chhabra et al. 1990
Maus, B6C3F1, 10 ♂, 10 ♀	13 Wo, 0, 66, 200, 600, 1800, 5000 ml/m^3^, 6 h/d, 5 d/Wo, Reinheit: 99 %, Peroxidgehalt: < 1,5 mg/l	**ab 200 ml/m^3^**: ♂: rel. Gewicht Leber** **↑; **ab 600 ml/m^3^**: ♂: abs. Gewicht Leber ↑, abs. und rel. Gewicht Thymusdrüse ↓; **ab 1800 ml/m^3^**: ** LOAEC**, ♂, ♀: Narkose, ♀: abs. und rel. Gewicht Leber ↑, Leber: zentrilobuläre Zytomegalie; **bei 5000 ml/m^3^**: ♂, ♀: Stupor 2 h lang nach Exposition, abs. und rel. Gewicht Milz ↓, Leber: zentrilobuläre Zytomegalie, ♂: KG ↓, ♀: abs. und rel. Gewicht Leber ↑, abs. und rel. Gewichte Lunge, Herz ↓, Nebennierenrinde: Degenerationen, Uterus: Atrophien	Chhabra et al. 1990
Meerschweinchen, 2 (k. w. A.)	20 d, 0, 10 000 mg/m^3^ (3330 ml/m^3^), 8 h/d, Reinheit: k. A.	leichte Narkose, Atemwegsschleimhaut: Reizungen (k. w. A.) (1/2), Niere: leichte Schäden (k. w. A.)	DuPont 2010
Katze, 4 (k. w. A.)	20 d, 0, 10 000 mg/m^3^ (3330 ml/m^3^), 8 h/d, Reinheit: k. A.	Mortalität: 2/4, leichte Narkose, Blut: erhöhter Gesamtproteingehalt (nach 4 Expositionen), Schleimhautreizungen: Speichel-, Tränenfluss, Nasenausfluss, verstärktes Blinzeln	DuPont 2010

abs.: absolut; AST: Aspartat-Aminotransferase; d: Tag; h: Stunde; rel.: relativ; Wo: Woche

a) Tetrahydrofuran kann in Gegenwart von Licht und Sauerstoff Peroxide bilden. Falls deren Gehalt in der Testsubstanz gemessen wurde, ist dieser angegeben.

#### 5.2.2 Orale Aufnahme

Seit der Begründung von 2003 (Greim 2003) ist folgende Studie neu hinzugekommen:

In einer Untersuchung nach OECD-Prüfrichtlinie 407 wurden jeweils zehn weibliche und männliche Sprague-Dawley-Ratten (jung, adult, k. w. A.) pro Dosisgruppe vier Wochen lang gegen 0, 1, 10, 100 oder 1000 mg Tetrahydrofuran/l im Trinkwasser exponiert. Dies entsprach Dosen von 0; 0,1; 0,8; 10,5 oder 95,5 mg/kg KG und Tag für männliche und 0,1; 1,0; 10,7 und 111,3 mg/kg KG und Tag für weibliche Ratten. Die Reinheit der Testsubstanz war 99,5 %. Es wurden keine Effekte auf Futteraufnahme, Körpergewicht oder Blutparameter festgestellt. Histopathologische Untersuchungen wiesen minimale bis leichte Veränderungen in Schilddrüse, Leber und Nieren nach Exposition gegen die höchste Dosis auf. In der Schilddrüse waren dies eine Verdickung des Epithels und eine reduzierte Dichte des Gewebes, in der Leber zytoplasmatische Veränderungen im perivenösen Bereich und in der Niere Tubulusveränderungen (u. a. eosinophile Einschlüsse, Pyknosen). Die Veränderungen wurden als adaptiv charakterisiert. Als NOAEL wurde ein Wert von 1000 mg Tetrahydrofuran/l Trinkwasser (111,3 mg Tetrahydrofuran/kg KG und Tag) angegeben (ECHA 2022).

#### 5.2.3 Dermale Aufnahme

Hierzu liegen keine Daten vor.

#### 5.2.4 Fazit

Wie schon nach akuter Exposition von Kaninchen ist nach wiederholter Exposition von Ratten der empfindlichste Endpunkt die Reizwirkung von Tetrahydrofuran und nachfolgende Zellveränderungen der nasalen und trachealen Mukosa. Die LOAEC für die Erniedrigung der Zilienschlagfrequenz und morphologische Schäden nach 3- und 12-wöchiger Exposition beträgt bei Ratten 100 bzw. 200 ml/m^3^ (Ohashi et al. 1982 a, b). In weiteren subchronischen Studien mit Ratten (Kawata und Ito 1984; Malley et al. 2001) sowie chronischen Studien mit Ratten und Mäusen bzw. subakuten Studien mit Katzen (Chhabra et al. 1990; DuPont 2010; NTP 1998) wurden keine Messung der Zilienschlagfrequenz oder elektronenmikroskopische Untersuchungen der nasalen und trachealen Schleimhaut vorgenommen. Aufgrund der sensitiven Methodik der Erfassung mittels Elektronenmikroskopie (Ohashi et al. 1982 a, b) konnte aber dieser adverse Effekt bei Ratten schon in geringer Ausprägung festgestellt werden. Eine Beteiligung von in der Testsubstanz enthaltenen Peroxiden an den beschriebenen Effekten im Atemtrakt ist wegen des geringen Gehalts nicht anzunehmen.

### 5.3 Wirkung auf Haut und Schleimhäute

#### 5.3.1 Haut

Bisher liegt die Information vor, dass flüssiges Tetrahydrofuran hautreizend wirken kann (Greim 2003).

Folgende Publikationen sind neu hinzugekommen:

Nach okklusiver Auftragung von 0,5 ml Tetrahydrofuran (Draize-Test, k. A. zur Reinheit der Testsubstanz) auf die rasierte intakte oder abradierte Haut von sechs Weiße-Neuseeländer-Kaninchen (männliche und weibliche, k. w. A.) für 24 Stunden wurden die Tiere 24 und 72 Stunden nach Applikation untersucht. Es wurde ein Irritationsindex von 1,93 errechnet. Nach 72 Stunden wurden noch leichte Ödeme und Erytheme festgestellt (Reizwerte intakte Haut, Erytheme: 4 × 1, 2 × 0 von maximal 4; Ödeme: jeweils 2 × 2, 1 bzw. 0 von maximal 4). Tetrahydrofuran wurde als leicht hautreizend beschrieben (GAF Corporation 1978). In der Studie wurden die Zeitpunkte 48 Stunden und 14 Tage nach der Applikation nicht untersucht, was die Aussagekraft einschränkt. Eine Aussage zur möglichen Reversibilität der Ödeme und Erytheme ist aufgrund des fehlenden Untersuchungszeitpunktes von 14 Tagen nicht zu treffen.

Nach Auftragung von 1 ml Tetrahydrofuran (k. A. zur Reinheit der Testsubstanz) auf die Haut von 20 Mäusen (k. w. A.) und Kaninchen (k. w. A.) wurden Rötungen und Hautablösungen festgestellt (US EPA 2012). Aufgrund fehlender oder unzureichender Angaben zur Testsubstanz, der Art der Auftragung und der Applikationsdauer sowie der Befunde ist diese Studie nicht für eine Bewertung geeignet.

In einer Studie zur dermalen Toxizität aus dem Jahr 2009 nach OECD-Prüfrichtlinie 402, wurden je fünf männliche und weibliche Wistar-Ratten 24 Stunden lang semi-okklusiv gegen 2000 mg Tetrahydrofuran/kg KG exponiert. Die Reinheit der Testsubstanz war > 99 %. Es wurden 30 bis 60 Minuten sowie sieben Tage nach Behandlungsende keine Hautreizungen durch Tetrahydrofuran festgestellt (ECHA 2022). 

**Fazit**: In den vorliegenden Studien, wobei eine nach OECD-Prüfrichtlinie 402 durchgeführt wurde, zeigt Tetrahydrofuran eine leichte hautreizende Wirkung bei Kaninchen und keine Reizwirkung an der Rattenhaut.

#### 5.3.2 Auge

Sechs Albino-Kaninchen wurde jeweils 0,1 ml Tetrahydrofuran (k. A. zur Reinheit der Testsubstanz) in den Bindehautsack des rechten Auges appliziert, wobei das linke Auge als Kontrolle diente. Die Augen der Tiere wurden 24, 48, 72 Stunden und 14 Tage nach Applikation untersucht. Bei zwei der sechs Kaninchen traten schwere und irreversible Hornhautschädigungen auf. Augenreizungen in Form von Rötungen bzw. Iritis traten bei allen Tieren auf und waren nach 14 Tagen noch bei drei von sechs Tieren (Rötung) oder nicht mehr feststellbar (Iritis) (DuPont 1971).

Sechs Neuseeländer-Kaninchen wurde 0,1 ml Tetrahydrofuran (k. A. zur Reinheit der Testsubstanz) in den Bindehautsack des rechten Auges appliziert, wobei das linke Auge als Kontrolle diente. Die Augen der Tiere wurden nicht gespült und 24 Stunden, zwei, drei, vier und sieben Tage nach der Applikation untersucht. Bei drei der Tiere wurde in den ersten 3–4 Tagen nach Applikation eine Bleichung der Bindehaut festgestellt. Der höchste mittlere Reizindex wurde 48 Stunden nach der Applikation beobachtet und betrug 31,2 von maximal 110. Zwei der Tiere zeigten auch sieben Tage nach der Applikation Rötung und Ödeme der Konjunktiven, Hornhauttrübung sowie fibrovaskuläres Bindegewebe. Tetrahydrofuran wurde als mäßig reizend am Kaninchenauge bewertet (GAF Corporation 1978).

**Fazit**: Tetrahydrofuran verursacht schwere und irreversible Augenschäden. Wirkungen auf die Schleimhaut des Atemtraktes sind in Abschnitt 5.2 beschrieben.

### 5.4 Allergene Wirkung

Bisher lagen keine Daten zur allergenen Wirkung beim Tier vor (Greim 2003).

#### 5.4.1 Hautsensibilisierende Wirkung

In einem Local Lymph Node Assay (LLNA) nach Kimber et al. (1989), ähnlich wie OECD-Prüfrichtlinie 429, zeigte Tetrahydrofuran (Reinheit 99,9 %) kein hautsensibilisierendes Potential. Getestet wurden Konzentrationen von 10, 25 oder 50 % in Ethanol an jeweils vier weiblichen CBA/Ca-Mäusen, wobei Stimulationsindices von 0,6; 0,7 und 0,7 bestimmt wurden. Damit ist das Ergebnis negativ (ECHA 2022).

In zwei weiteren, analog der OECD-Prüfrichtlinie 429 durchgeführten LLNA an jeweils vier weiblichen CBA/Ca-Mäusen wurde getestet, ob Tetrahydrofuran als Lösungsmittel geeignet ist. Die Studienergebnisse wurden ebenfalls als negativ bewertet. Es wurden jedoch keine Stimulationsindices berechnet. Auf Basis des Vergleichs der erhaltenen Zerfälle pro Minute (DPM) mit Werten anderer, bekanntermaßen nicht sensibilisierenden Substanzen, die als Lösungsmittel (k. w. A.) im LLNA eingesetzt werden, wurde Tetrahydrofuran als nicht sensibilisierend bewertet (ECHA 2022). 

#### 5.4.2 Atemwegssensibilisierende Wirkung

Hierzu liegen keine Untersuchungen vor.

### 5.5 Reproduktionstoxizität

Seit Erscheinen der Begründung (Greim 2003) liegen keine neuen Daten zu diesem Endpunkt vor. Aufgrund der neuen Bewertung des MAK-Werts wird im Rahmen dieses Nachtrags der Endpunkt Reproduktionstoxizität erneut überprüft und evaluiert. 

#### 5.5.1 Fertilität

In einer Ein-Generationen-Dosisfindungsstudie wurde Tetrahydrofuran je zehn männlichen und weiblichen Wistar-Ratten pro Gruppe in Konzentrationen von 0, 4000, 8000 oder 12 000 mg/l Trinkwasser (entspricht mittleren Dosierungen für die männlichen Tiere von 0, 444, 796, 1107 mg/kg KG und Tag; für die weiblichen Tiere vor der Verpaarung von 0, 467, 798, 1088 mg/kg KG und Tag bzw. während der Gestation von 0, 434, 758, 1139 mg/kg KG und Tag) sieben Wochen vor und während der Verpaarung, sowie während Trächtigkeit und Laktation verabreicht. Bei der höchsten Dosierung kam es ausschließlich bei männlichen Tieren zu einer reduzierten Futteraufnahme. Die relativen Nierengewichte waren bei weiblichen Ratten ab 8000 mg/l und bei männlichen Tieren bei 12 000 mg/l erhöht (BASF AG 1994). 

In der anschließenden Zwei-Generationenstudie nach OECD-Prüfrichtlinie 416 erhielten 25 Wistar-Ratten pro Geschlecht und Dosis 0, 1000, 3000 oder 9000 mg Tetrahydrofuran/l Trinkwasser (entspricht für die F0-Generation: männliche Tiere 0, 91, 268, 714 mg/kg KG und Tag; weibliche Tiere vor der Verpaarung: 0, 104, 301, 742 mg/kg KG und Tag; während der Gestation: 0, 104, 288, 790 mg/kg KG und Tag) 70 Tage vor und während der Verpaarung sowie während Gestation und Laktation. Bei der höchsten Dosierung kam es zu deutlicher Toxizität bei den Elterntieren in Form von reduzierter Wasser- und Futteraufnahme sowie reduzierter Körpergewichtsentwicklung. Bei den männlichen und weiblichen Tieren der F0-Generation war das Nierengewicht statistisch signifikant erhöht, jedoch ohne histopathologisches Korrelat. Somit liegt der NOAEL für Parentaltoxizität bei 3000 mg/l, entsprechend für die männlichen Tiere 268 mg/kg KG und Tag und für die weiblichen Tiere ca. 295 mg/kg KG und Tag. Weder klinisch noch histopathologisch gab es Hinweise auf eine Beeinträchtigung der Fertilität und des Reproduktionsvermögens der Tiere, sodass der NOAEL für Fertilität 9000 mg Tetrahydrofuran/l (für die männlichen Tiere 714 mg/kg KG und Tag und für die weiblichen Tiere ca. 742 mg/kg KG und Tag) beträgt (BASF AG 1996; Hellwig et al. 2002).

#### 5.5.2 Entwicklungstoxizität

In der Begründung aus dem Jahr 2003 (Greim 2003) werden Studien zur pränatalen Entwicklungstoxizität nach OECD-Prüfrichtlinie 414 an Sprague-Dawley-Ratten und Swiss-CD1-Mäusen mit inhalativer Ganzkörperexposition vom 6. bis zum 19. bzw. 6. bis zum 17. Gestationstag täglich sechs Stunden lang gegen nominale Konzentrationen von 0, 600, 1800 oder 5000 ml/m^3^ beschrieben. Die analytischen Konzentrationen lagen für die Ratten bei 0 (gefilterte Luft), 610, 1830 und 5060 ml/m^3^ und für die Mäuse bei 0, 598, 1800, 4990 ml/m^3^. Bei Ratten kam es bei der höchsten Konzentration zu erniedrigten Körpergewichten der Feten bei gleichzeitiger Maternaltoxizität in Form einer verringerten Körpergewichtszunahme. Bei Mäusen war bei 1800 ml/m^3^ die Anzahl lebender Feten/Wurf erniedrigt (9,3 ± 4,4; Kontrolle: 11,9 ± 1,9) bei gleichzeitiger Maternaltoxizität (Sedierung, erniedrigtes Uterusgewicht). Die höchste Konzentration erwies sich als deutlich maternaltoxisch. So trat bei allen Tieren Sedierung mit einer hohen Mortalität von 27 % auf, was zum Expositionsabbruch am 12. Gestationstag führte. Die NOAEC für Maternal- und Entwicklungstoxizität lagen damit bei 1830 ml/m^3^ für Sprague-Dawley-Ratten und 598 ml/m^3^ für Swiss-CD1-Mäuse. Weder bei Ratten noch bei Mäusen traten teratogene Effekte auf (Greim 2003; Mast et al. 1992).

Folgende Studien sind neu hinzugekommen:

In einer Vorstudie zur Entwicklungstoxizität wurden je sieben weibliche Crl:CD-Ratten sechs Stunden pro Tag gegen Tetrahydrofuran (Reinheit > 99,9 %) vom 6. bis zum 15. Gestationstag (Untersuchung am 21. Gestationstag) Ganzkörper-exponiert. Die Tiere wurden gegen analytische Konzentrationen von 0 (gefilterte Luft), 213, 520, 2470 oder 4940 ml/m^3^ exponiert. Bei den Feten kam es bei 4940 ml/m^3^ zu erniedrigtem Körpergewicht und petechialen Hämorrhagien. Maternale Toxizität trat ab 2470 ml/m^3^ in Form einer reduzierten Antwort auf einen Lärmstimulus sowie Chromodakryorrhoe und Chromorhinorrhoe auf. Bei der höchsten Konzentration zeigten die Muttertiere eine erniedrigte Körpergewichtszunahme und Futteraufnahme, Lethargie, einen geringen Muskeltonus, taumelnden Gang und Alopezie (Haskell Laboratories 1980).

In der nachfolgenden Studie zur pränatalen Entwicklungstoxizität ähnlich der OECD-Prüfrichtlinie 414 an Crl:CD-Ratten mit dem gleichen Applikationsschema wurden jeweils 27 weibliche Tiere gegen analytische Konzentrationen von 0 (gefilterte Luft), 1002 oder 4934 ml/m^3^ exponiert. Bei 4934 ml/m^3^ kam es bei den Feten zu erniedrigten Körpergewichten und einer verzögerten Ossifikation der Sternebrae. Teratogene Effekte traten nicht auf. Die Muttertiere wiesen ab der niedrigsten Konzentration eine reduzierte Antwort auf einen Lärmstimulus auf, und bei 4934 ml/m^3^ kam es zu erniedrigter Körpergewichtszunahme und Futteraufnahme, Lethargie und taumelndem Gang (Haskell Laboratories 1982). Die NOAEC für Entwicklungstoxizität lag bei 1002 ml/m^3^. Eine NOAEC für Maternaltoxizität konnte nicht abgeleitet werden.

In den bereits beschriebenen Generationenstudien, der Ein-Generationen-Dosisfindungsstudie und der anschließenden Zwei-Generationenstudie, kam es bis zu den jeweils höchsten Dosierungen bis zum 4. Postnataltag nicht zu Effekten auf die Nachkommen (siehe Abschnitt 5.5.1; BASF AG 1994; Greim 2003; Hellwig et al. 2002). Daraus leiten sich NOAEL für Perinataltoxizität von 1139 bzw. 790 mg/kg KG und Tag ab. 

**Fazit**: Weder bei Ratten noch bei Mäusen wurden teratogene Effekte beobachtet. In zwei Studien zur pränatalen Entwicklungstoxizität nach OECD-Prüfrichtlinie 414 an Sprague-Dawley-Ratten bzw. Crl:CD-Ratten mit Ganzkörperexposition (sechs Stunden pro Tag) traten bei gleichzeitiger Maternaltoxizität (erniedrigte Körpergewichtszunahme und Futteraufnahme, Lethargie und taumelnder Gang) bei 5060 ml/m^3^ reduzierte Fetengewichte bzw. bei 4934 ml/m^3^ erniedrigte Körpergewichte und eine verzögerte Ossifikation der Sternebrae auf. Die NOAEC für Entwicklungstoxizität lag bei 1830 bzw. 1002 ml/m^3^. In der ersten Studie lag die NOAEC für Maternaltoxizität bei 1830 ml/m^3^, in der zweiten Studie war 1002 ml/m^3^ eine LOAEC für Maternaltoxizität.

Bei Swiss-CD1-Mäusen war in einer Ganzkörperinhalationsstudie (sechs Stunden pro Tag) zur pränatalen Entwicklungstoxizität nach OECD-Prüfrichtlinie 414 bei 1800 ml/m^3^ die Anzahl lebender Feten/Wurf bei gleichzeitiger maternaler Toxizität (sedierende Effekte, erniedrigtes Uterusgewicht) erniedrigt. Die NOAEC für Maternal- und Entwicklungstoxizität lag bei 598 ml/m^3^ für Swiss-CD1-Mäuse. Aus einer 2-Generationenstudie nach OECD-Prüfrichtlinie 416 an Ratten leitet sich ein NOAEL für Perinataltoxizität von 790 mg/kg KG und Tag ab, hier kam es bis zu dieser höchsten Dosierung bei gleichzeitiger Maternaltoxizität bis zum 4. Postnataltag nicht zu Effekten auf die Nachkommen. 

### 5.6 Genotoxizität

In den in der Begründung aus dem Jahr 2003 (Greim 2003) aufgeführten Studien war Tetrahydrofuran nicht genotoxisch. Es wurden in vitro in Bakterien keine Mutationen induziert und in Säugerzellen keine Schwesterchromatidaustausche, Chromosomenaberrationen oder Mikronuklei. In vivo führte die Behandlung mit Tetrahydrofuran bei Mäusen nicht zu vermehrten Schwesterchromatidaustauschen oder Chromosomenaberrationen im Knochenmark und auch nicht zu gesteigerter UDS in der Leber nach intraperitonealer Gabe. Inhalative Exposition induzierte keine Mikronuklei im Knochenmark bei Mäusen (Greim 2003). Seit dieser Publikation sind die folgend beschriebenen Studien hinzugekommen:

#### 5.6.1 In vitro

##### 5.6.1.1 Zellfreie Systeme

Um die Wirkung von Photooxidationsprodukten auf Nukleoside zu untersuchen, wurde Tetrahydrofuran mittels Licht oxidiert und mit Kalbsthymus-DNA oder mit den Nukleosiden 2′-Desoxyguanosin (dGuo), 2′-Desoxycytidin (dCyd) bzw. 2′-Desoxyadenosin (dAdo) inkubiert. Bei der Photooxidation entstanden 2-Hydroxytetrahydrofuran und verschiedene Peroxide. Mittels NMR oder massenspektrometrischer Analyse wurden vier Addukte identifiziert: 9-(4-Hydroxy-5-hydroxymethyloxolan-2-yl)-2-(oxolan-2-ylamino)-1,9-dihydropurin-6-on (dGuo-THF1), 2-Amino-9-(4-hydroxy-5-hydroxymethyloxolan-2-yl)-1-(oxolan-2-yl)-1,9-dihydropurin-6-on (dGuo-THF2), 1-(4-Hydroxy-5-hydroxymethyloxolan-2-yl)-4-(oxolan-2-ylamino)-1H-pyrimidin-2-on (dCyd-THF) und 2-Hydroxymethyl-5-[6-(oxolan-2-ylamino)-purin-9-yl]-oxolan-3-ol (dAdo-THF). Bei Inkubation von nicht oxidiertem Tetrahydrofuran mit dGuo in Anwesenheit von Rattenleber-Mikrosomen und NADPH wurde ebenfalls das Addukt dGuo-THF1 identifiziert. Zusätzlich war die Konzentration von 8-Oxo-Desoxyguanosin (8-Oxo-dGuo) nach Zugabe von Tetrahydrofuran statistisch signifikant vermindert, was die Autoren auf eine Kompetition von dGuo und Tetrahydrofuran an den aktiven Zentren der mikrosomalen Enzyme zurückführten (Hermida et al. 2006). Die Stabilität der Addukte, außer dGuo-THF2, war nur nach Reduktion mittels Natriumborhydrid gewährleistet und es wurden hohe Mengen an Tetrahydrofuran in die Ansätze eingebracht. Zudem ist die Aussage der 8-Oxo-dGuo-Detektion fragwürdig, da im zellulären System eine Kompartimentierung zwischen DNA und mikrosomalen Enzymen besteht. 

In einer weiteren Studie wurde der Einfluss von Tetrahydrofuran auf die Bildung endogener Etheno-DNA-Addukte, die vermutlich auf Lipidperoxidation zurückgehen, untersucht. Dazu wurden bei der Lipidperoxidation gebildete ungesättigte Aldehyde in Gegenwart von dGuo und oxidiertem oder nicht oxidiertem Tetrahydrofuran inkubiert. Nur mit oxidiertem Tetrahydrofuran bildeten sich drei Addukte: 3-(2′-Desoxy-β-D-erythropentafuranosyl)-7-[3-hydroxy-1-(3-(2′-deoxy-β-D-erythropentafuranosyl)-3,5-dihydroimidazo-[1,2-a]purin-9-on-7-yl)propyl]-3,5-dihydro-imidazo[1,2-a]purin-9-on sowie zwei Isomerformen des 3-(2′-Desoxy-β-D-erythropentafuranosyl)-7-(tetrahydrofuran-2-yl)-3,5-dihydroimidazo[1,2-a]purin-9-ons. Die Adduktbildung beruhte auf einer Reaktion von 4-Hydroxybutanal, gebildet nach Ringöffnung von hydroxyliertem Tetrahydrofuran, mit dem zuvor gebildeten Etheno-DNA-Addukt 1,N2-εdGuo (Loureiro et al. 2005).

##### 5.6.1.2 Bakterien

In einer nicht im Original vorliegenden Rückmutationsstudie mit Escherichia coli Wp2 uvrA wurden mit 1 µmol Tetrahydrofuran/l 10 % der Effekte hervorgerufen, die durch die Positivkontrolle (Epibromhydrin) induziert wurden. Relevante Angaben zur Versuchsdurchführung liegen nicht vor (RAC 2009).

In einer Studie zur Untersuchung des mutagenen Potentials von Frittierölen wurde Tetrahydrofuran als Lösungsmittelkontrolle eingesetzt. Tetrahydrofuran war in An- und Abwesenheit eines metabolischen Aktivierungssystems nicht mutagen in den Salmonella-typhimurium-Stämmen TA97, TA100 und TA104 (Hageman et al. 1988).

In Vorversuchen zu einer weiteren Studie erwies sich Tetrahydrofuran als Lösungsmittelkontrolle in einem Platteninkorporationstest bei einer Konzentration von 50 µl/Platte als nicht mutagen in Salmonella typhimurium TA100. Höhere Konzentrationen wirkten zytotoxisch (Maron et al. 1981). 

Die Ergebnisse der beiden letzten Studien sind für eine Bewertung nicht geeignet, da Tetrahydrofuran als Lösungsmittelkontrolle eingesetzt wurde und die Ergebnisse nicht mit unbehandelten Kontrollen verglichen wurden. 

##### 5.6.1.3 Säugerzellen

In einem HPRT-Lokustest nach OECD-Prüfrichtlinie 476 wurde keine mutagene Wirkung in CHO-Zellen nach Exposition gegen Tetrahydrofuran in Konzentrationen von 0; 93,8; 187,5; 250; 375; 500 oder 750 µg Tetrahydrofuran/ml in An- oder Abwesenheit eines metabolischen Aktivierungssystems beobachtet. Es wurde bei keiner der eingesetzten Konzentrationen eine zytotoxische Wirkung von Tetrahydrofuran festgestellt (ECHA 2022). 

#### 5.6.2 In vivo

Es liegen keine neuen Daten vor.

#### 5.6.3 Fazit

Neue Untersuchungen in zellfreien, artifiziellen Systemen liefern Hinweise auf die Bildung möglicher DNA-Addukte aufgrund elektrophiler Intermediate des Tetrahydrofurans nach Photooxidation. Entsprechende Belege für die Adduktbildung in vivo liegen aber nicht vor. Dies ist damit zu begründen, dass die Addukte unter In-vivo-Bedingungen instabil sind, da sie in den In-vitro-Versuchen nur durch eine vorherige Reduktion mittels Natriumborhydrid stabilisiert werden konnten. Zudem wurde die Adduktbildung in vitro nur bei Inkubation mit hohen Konzentrationen von Tetrahydrofuran erzielt. 

Ein HPRT-Lokustest zeigt kein mutagenes Potential in Hamsterzellen auf. Zusammen mit den bereits in der Begründung von 2003 (Greim 2003) dargestellten Untersuchungen betrachtet wirkt Tetrahydrofuran in vitro und in vivo nicht genotoxisch.

### 5.7 Kanzerogenität

#### 5.7.1 Kurzzeitstudien

Tetrahydrofuran erwies sich in zwei Zelltransformationstests an BALB/c-3T3-Zellen und Embryozellen des Syrischen Goldhamsters als nicht transformierend und als nicht initiierend an der Mäusehaut (Greim 2003). 

Folgende Studien sind neu hinzugekommen:

Für eine Untersuchung zur Tumorinduktion wurden Zellen der N-Zelllinie B/C-N 7.1 mittels Benzo[a]pyrenepoxid transformiert, wobei Tetrahydrofuran als Lösungsmittel diente. Zur Kontrolle wurden auch Zellen nur gegen Tetrahydrofuran exponiert. Die Zellen wurden anschließend in zwei Mausstämme injiziert. Zellen aus 2/20 Ansätzen mit Tetrahydrofuran induzierten Tumoren, wohingegen 46/57 Ansätzen mit Benzo[a]pyrenepoxid tumorinduzierend waren (k. w. A.) (Collins et al. 1982). Die Inzidenz für unbehandelte Mäuse wurde nicht berichtet. Die Studie ist daher nicht für eine Bewertung von Tetrahydrofuran geeignet.

In einer nur als Abstract veröffentlichten Studie wurden embryonale Zellen des Syrischen Goldhamsters zwei oder 20 Stunden gegen Tetrahydrofuran in der Gasphase in geschlossenen Kammern exponiert (k. w. A.). Nach Messung der Überlebensrate wurde eine gesteigerte Transformationsrate der DNA der Zellen durch das SA7 Adenovirus festgestellt (k. w. A.) (RAC 2009). Da relevante Angaben zu Versuchsdurchführung und Ergebnissen nicht vorliegen, ist diese Studie nicht für eine Bewertung von Tetrahydrofuran geeignet. 

#### 5.7.2 Langzeitstudien

Die Ergebnisse aus Langzeitversuchen des NTP wurden in der Begründung von 2003 (Greim 2003) beschrieben. Da zu diesen Studien neue Nachuntersuchungen sowie publizierte Bewertungen vorliegen (Bruner et al. 2010; Dekant 2019; Fenner-Crisp et al. 2011) sind im Folgenden die Ergebnisse erneut dargestellt.

##### Nierenadenome und Neoplasien bei männlichen F344-Ratten

In einer 2-Jahre-Kanzerogenitätsstudie wurden je 50 F344-Ratten und B6C3F1-Mäuse beiderlei Geschlechts sechs Stunden pro Tag an fünf Tagen pro Woche gegen 0, 200, 600 oder 1800 ml Tetrahydrofuran/m^3^ exponiert. Bei 0, 200, 600 oder 1800 ml/m^3^ betrugen die kombinierten Inzidenzen von Nierenadenomen und -karzinomen 1/50, 1/50, 4/50 bzw. 5/50 bei männlichen Ratten. Sie waren im Gruppenvergleich nicht statistisch signifikant erhöht, jedoch war der Trend-Test positiv (p = 0,037). Auch bei möglichen Vorstufen (Tubulushyperplasien) ergaben sich keine statistisch signifikant erhöhten Inzidenzen. Die Mortalität der exponierten männlichen Ratten war erhöht, aber im Vergleich zur Kontrolle nicht statistisch signifikant. Im Vergleich zu historischen Kontrollen des NTP (Studiendauer 729–735 Tage) war die Mortalität der F344-Ratten nicht höher als in anderen Inhalationsstudien des NTP. Bei nahezu allen Ratten, inklusive der Kontrolle, entwickelte sich eine CPN (NTP 1998), die bei diesem Rattenstamm in 2-Jahre-Kanzerogenitätsstudien des NTP häufig auftritt (Hard et al. 2012).

In einer pathologischen Untersuchung, die als persönliche Mitteilung von Hard in Fenner-Crisp et al. (2011) zitiert ist, wurden die Nierenschnitte der männlichen und weiblichen Ratten der Kontrollgruppe und der Tiere, die gegen 1800 ml/m^3^ exponiert waren, erneut untersucht. Aus den Gruppen, die gegen 200 oder 600 ml/m^3^ exponiert waren, wurden die Nierenschnitte der männlichen Ratten untersucht, bei denen, laut Auswertung des NTP, renale tubuläre Hyperplasien, Adenome oder Karzinome festgestellt wurden. Folgende Befunde wurden unterschiedlich im Vergleich zur NTP-Auswertung bewertet: eine renale tubuläre Hyperplasie in der Kontrollgruppe wurde als Adenom eingestuft, ein Adenom in der 600-ml/m^3^-Gruppe als atypische tubuläre Hyperplasie und die zwei Karzinome in der 1800-ml/m^3^-Gruppe als Adenome. Somit ergaben sich in dieser Studie Tumorinzidenzen von 2/50, 1/50, 3/50 und 5/50 bei Exposition gegen 0, 200, 600 bzw. 1800 ml/m^3^. Alle Tumoren wurden als Adenome charakterisiert. 

Eine weitere histopathologische Nachuntersuchung (Bruner et al. 2010) erfolgte mit Nierenschnitten aus drei Studien, die bereits in Greim (2003) beschrieben wurden: der 4-Wochen-Studie von Gamer et al. (2002), der 13-Wochen-Inhalationsstudie (Chhabra et al. 1990; NTP 1998) und der 2-Jahre-Kanzerogenitätsstudie (NTP 1998). 

Aus der 13-Wochen- und der 4-Wochen-Studie wurden alle verfügbaren Nierenschnitte erneut untersucht. Die Nachuntersuchung der 2-Jahre-Kanzerogenitätsstudie erfolgte mit Schnitten aller männlichen Ratten der Kontrollgruppe, der 1800-ml/m^3^-Gruppe und Schnitten aus den Gruppen, die gegen 200 oder 600 ml/m^3^ exponiert waren und bei denen zuvor proliferative Läsionen beobachtet worden waren. In der Nachuntersuchung wurde ebenfalls der Schweregrad der CPN bewertet, wobei Grad 1, 2, 3 und 4 eine minimale, milde, moderate bzw. schwere CPN beschreibt. 

Die Untersuchung der Schnitte aus der 4-Wochen-Studie von Gamer et al. (2002) bestätigte den Befund einer statistisch signifikant erhöhten Inzidenz hyaliner Tröpfchen in den proximalen Tubuluszellen männlicher Ratten (exponiert gegen 1800 ml/m^3^). Ebenso wurde der Befund bestätigt, dass eine erhöhte Zellproliferation im Nierenkortex der Ratten nach 20-tägiger Exposition gegen 1800 ml/m^3^ stattfand. Es wurde kein Anzeichen einer Zytotoxizität in den Schnitten festgestellt. 

Die Nachuntersuchung der Schnitte der 14-Wochen-Studie bestätigte den Befund hyaliner Tröpfchen im proximalen Tubulusepithel der männlichen Ratten in der 5000-ml/m^3^-Gruppe, der Beleg für einen α-2u-Globulin-assoziierten Mechanismus sein kann. Es wurden jedoch ähnliche Befunde in der Kontrollgruppe festgestellt und aufgrund der Färbung der Schnitte war eine weitere Unterscheidung nicht möglich. Weitere Untersuchungen zur histologischen Bestätigung eines α-2u-Globulin-assoziierten Mechanismus ergaben, dass granuläre Zylinder von exfoliierten Tubuluszellen an der Verbindungsstelle der äußeren und inneren Streifen der äußeren Medulla und eine vermehrte Anzahl hyaliner Tröpfchen oder ihre Größenzunahme nicht beobachtet wurden. 

Die Untersuchung der Schnitte der 2-Jahre-Kanzerogenitätsstudie bestätigte die bereits in der ersten Nachuntersuchung (Hard in Fenner-Crisp et al. 2011) getroffene Schlussfolgerung, dass alle Tumoren als Adenome zu charakterisieren sind. Es ergaben sich Inzidenzen von Adenomen von 2/50, 0/50, 2/50 und 7/50 in der Kontroll-, 200-, 600- bzw. 1800-ml/m^3^-Gruppe. Alle Adenome und atypischen tubulären Hyperplasien traten bei Stadien schwerer CPN bzw. in ihrem Endstadium auf. Laut der Autoren ist die tubuläre Zelldegeneration und Regeneration in Folge der CPN möglicherweise verantwortlich für die Entwicklung der proliferativen Läsionen. Eine Verstärkung der CPN ist aber nicht belegt, da die mittleren Schweregrade der CPN bei Kontrolltieren und Tieren der höchsten Konzentrationsgruppe ähnlich waren. Im Rahmen dieser Bewertung wurden nur atypische Tubulushyperplasien als präneoplastisch definiert. Insgesamt wurde bei 1800 ml/m^3^ kein statistisch signifikanter Anstieg der Inzidenz der Summe von präneoplastischen und benignen neoplastischen Läsionen festgestellt. Die Anzahl präneoplastischer Läsionen (atypische Hyperplasien) war in der Kontrollgruppe höher als bei den historischen Kontrollen (Hard et al. 2012). Aufgrund der ähnlichen Inzidenz und Schweregrade der CPN bei männlichen Kontrolltieren und der männlichen Tiere in der höchsten Konzentrationsgruppe, ist kein kausaler Zusammenhang von Hyperplasien und Adenomen mit einer Exposition gegen Tetrahydrofuran belegt (Bruner et al. 2010).

Die Zusammenfassung der Befunde der Nachuntersuchungen von Nierenschnitten aus der 2-Jahre-Kanzerogenitätsstudie und der vormaligen Bewertung des NTP sind in Tabelle 2 dargestellt. 

##### Leberadenome und -karzinome in weiblichen B6C3F1-Mäusen

In der 2-Jahre-Kanzerogenitätsstudie wurden bei weiblichen Mäusen, die gegen die höchste Konzentration von 1800 ml Tetrahydrofuran/m^3^ exponiert waren, statistisch signifikant erhöhte Inzidenzen von Leberadenomen und -karzinomen festgestellt. Die Überlebensrate der männlichen Mäuse war bei 1800 ml/m^3^ statistisch signifikant verringert und 26 Tiere starben im ersten Jahr. Als Ursache wurde die narkotische Wirkung von Tetrahydrofuran bei dieser Konzentration und aufsteigende Harnwegsinfektionen, die durch das im Urogenitalbereich durchnässte Fell während der Narkose begünstigt wurden, diskutiert. Die Autoren schlussfolgern, dass somit 1800 ml/m^3^ über der maximal tolerierbaren Dosis für männliche Mäuse gelegen hat (NTP 1998).

Die Befunde wurden bereits wie folgt bewertet und in Tabelle 2 aufgeführt: Die mitogene Wirkung in der Leber weiblicher Mäuse trat bei Konzentrationen unterhalb und zeitlich vor der kanzerogenen Wirkung auf. Eine Konzentration von 200 ml/m^3^ stellt für die Erhöhung des Mitose-Index unter Berücksichtigung des biologischen Trends die LOAEC, unter Betrachtung der statistischen Signifikanz die NOAEC dar. Die Zellproliferationsrate zeigt ebenfalls einen konzentrationsabhängigen Verlauf mit einer NOAEC von 200 ml/m^3^ (biologischer Trend, aber statistisch nicht signifikant) bzw. einer LOAEC von 1800 ml/m^3^ (statistische Signifikanz). Eine statistisch signifikant verstärkte Lebernekrose bei den exponierten Tieren wurde im Vergleich zur Kontrolle nicht beobachtet und die Befunde zu Lebernekrosen waren nicht dosisabhängig (Greim 2003).

**Tab.2:** Studien zur Kanzerogenität von Tetrahydrofuran mit F344-Ratten und B6C3F1-Mäusen, inklusive der Befunde der Nachuntersuchung von Nierenschnitten

Autor:	NTP (1998)				
Stoff:	Tetrahydrofuran (Reinheit 99 %)				
Spezies:	**Ratte**, F344/N, 50 ♂, 50 ♀ pro Gruppe				
Applikation:	Inhalation				
Konzentration:	0, 200, 600, 1800 ml/m^3^				
Dauer:	2 a, 5 d/Wo, 6 h/d				
Toxizität:	–				
		**Konzentration [ml/m^3^]**			
		**0**	**200 **	**600 **	**1800**
Überlebende	♂	12/50 (24 %)	6/50 (12 %)	5/50 (10 %)	6/50 (12 %)
	♀	25/50 (50 %)	25/50 (50 %)	26/50 (52 %)	26/50 (52 %)
Durchschnittliche Überlebenszeit (d)	♂	616	626	627	627
	♀	657	665	685	671
**Tumoren und Präneoplasien**					
**Niere:**					
Hyperplasien	♂	7/50 (14 %)^a)^ 5/50 (10 %)^b)^ 7/50 (14 %)^c)^ 4/50 (8 %)^d)^	5/50 (10 %)^a)^ 3/50 (6 %)^b)^ 5/50 (10 %)^c)^ 2/50 (4 %)^d)^	6/50 (12 %)^a)^ 5/50 (10 %)^b)^ 6/50 (12 %)^c)^ 4/50 (8 %)^d)^	7/50 (14 %)^a)^ 6/50 (12 %)^b)^ 7/50 (14 %)^c)^ 1/50 (2 %)^d)^
Tubulusadenome	♂	1/50 (2 %)^a)^^, ^^#^ 2/50 (4 %)^b)^ 2/50 (4 %)^e)^	1/50 (2 %)^a)^ 1/50 (2 %)^b)^ 0/50^e)^	4/50 (8 %)^a)^ 3/50 (6 %)^b)^ 2/50 (4 %)^e)^	3/50 (6 %)^a)^ 5/50 (10 %)^b)^ 7/50 (14 %)^e)^
Tubuluskarzinome	♂	0/50^a), #^ 0/50^b)^ 0/50^e)^	0/50^a)^ 0/50^b)^ 0/50^e)^	0/50^a)^ 0/50^b)^ 0/50^e)^	2/50 (4 %)^a)^ 0/50^b)^ 0/50^e)^
erstes Auftreten eines Nierentumors (d)	♂	733	733	631	668
CPN (Schweregrad)	♂	48/50 (3,0^a)^) 49/50 (2,84^e)^)	50/50 (2,9^a)^)	50/50 (3,1^a)^)	50/50 (3,0^a)^) 50/50 (2,88^e)^)
Autor:	NTP (1998)				
Stoff:	Tetrahydrofuran (Reinheit 99 %)				
Spezies:	**Maus**, B6C3F1, 50 ♂, 50 ♀ pro Gruppe				
Applikation:	Inhalation				
Konzentration:	0, 200, 600, 1800 ml/m^3^				
Dauer:	2 a, 5 d/Wo, 6 h/d				
Toxizität:	1800 ml/m^3^: Mortalität ↑ aufgrund Narkose und bakterieller Infektion (♂)				
		**Konzentration [ml/m^3^]**			
		**0**	**200 **	**600 **	**1800**
Überlebende	♂	32/50 (64 %)	31/50 (62 %)	28/50 (56 %)	12/50 (24 %)*
	♀	29/50 (58 %)	33/50 (66 %)	26/50 (52 %)	32/50 (64 %)
**Tumoren und Präneoplasien**					
**Leber:**					
Lebernekrosen	♂	8/50 (16 %)	5/50 (10 %)	6/50 (12 %)	4/50 (8 %)
	♀	3/50 (6 %)	0/50 (0 %)	0/50 (0 %)	7/48 (15 %)
Adenome	♂	24/50 (48 %)	19/50 (38 %)	14/50 (28 %)	9/50 (18 %)
	♀	12/50 (24 %)	17/50 (38 %)	18/50 (36 %)	31/48 (65 %)*
Karzinome	♂	14/50 (28 %)	13/50 (26 %)	14/50 (28 %)	9/50 (18 %)
	♀	6/50 (14 %)	10/50 (20 %)	10/50 (20 %)	16/48 (33 %)*
Leberadenome/-karzinome	♂	33/50 (66 %)	31/50 (62 %)	30/50 (60 %)	18/50 (36 %)
	♀	17/50 (34 %)	24/50 (48 %)	26/50 (52 %)	41/48 (85 %)*

a: Jahr; CPN: chronisch progressive Nephropathie; d: Tag; h: Stunde; Wo: Woche

*p < 0,05;

# Trendtest (p = 0,037) für Summe von Adenomen und Karzinomen

a) Bewertung gemäß NTP (1998)

b) Bewertung gemäß Hard in Fenner-Crisp et al. (2011)

c) Bewertung gemäß Pathology Working Group in Fenner-Crisp et al. (2011)

d) Bewertung als atypische Hyperplasie gemäß Bruner et al. (2010)

e) Bewertung gemäß Bruner et al. (2010)

**Fazit**: In einer 2-Jahre-Kanzerogenitätsstudie wurde bei männlichen F344-Ratten bei der höchsten Konzentration von 1800 ml Tetrahydrofuran/m^3^ eine erhöhte Inzidenz von Nierenadenomen festgestellt. In der Gesamtschau der Datenlage ergeben sich Belege für einen α-2u-Globulin-assoziierten Mechanismus. Ein Zusammenhang der Inzidenz der atypischen Hyperplasien und Adenome bei männlichen F344-Kontroll-Ratten und dem Schweregrad einer CPN wurde aufgezeigt (Hard et al. 2012). Jedoch war im Fall von Tetrahydrofuran die Inzidenz und Schwere der CPN in der am höchsten exponierten Rattengruppe im Vergleich zur Kontrollgruppe ähnlich, so dass Tetrahydrofuran die CPN nicht verstärkte. Somit könnte die Entstehung der Adenome auf eine Kombinationswirkung von CPN und einem α-2u-Globulin-assoziierten Mechanismus zurückzuführen sein (siehe auch Abschnitt 2). Bei weiblichen B6C3F1-Mäusen wurden bei Exposition gegen Tetrahydrofuran statistisch signifikant erhöhte Inzidenzen von Leberadenomen und -karzinomen festgestellt, bei männlichen Mäusen trat eine erhöhte Mortalität auf, da hier die maximal tolerierbare Dosis überschritten war. Die Tumoren der weiblichen Mäuse entstehen als eine Folge einer schwachen CAR/PXR-Aktivierung durch Tetrahydrofuran mit den beschriebenen Folgeeffekten (siehe auch Abschnitt 2). 

## 6 Bewertung

Der empfindlichste Endpunkt ist die lokale Wirkung von Tetrahydrofuran auf die Nasen- und Tracheaschleimhaut bei Ratten und Kaninchen.

**MAK-Wert. **Tetrahydrofuran in einer Konzentration von 100 ml/m^3^ führte bei 3-wöchiger inhalativer Exposition von Ratten, vier Stunden pro Tag an fünf Tagen je Woche, zu einer Erniedrigung der Zilienschlagfrequenz und zu leichten Schädigungen des nasalen und trachealen Epithels (Ohashi et al. 1982 b). Nach 12-wöchiger Exposition kam es ab 200 ml Tetrahydrofuran/m^3^ zu leichten Schädigungen des nasalen und trachealen Epithels (Ohashi et al. 1982 a). Es tritt keine Verstärkung mit zunehmender Expositionsdauer ein.

Die systemische NOAEC ist 500 ml Tetrahydrofuran/m^3^ für narkotische Wirkung in einer 13-Wochen-Studie an Ratten. Zur toxikokinetischen Übertragung dieser Konzentration auf den Menschen werden berücksichtigt: die Übertragung der Daten des Tierversuchs auf den Menschen (1:2) und das erhöhte Atemvolumen am Arbeitsplatz (1:2). Die Narkosewirkung ist konzentrationsabhängig, wodurch keine Extrapolation auf eine chronische Exposition nötig ist. Daraus resultiert eine Konzentration von 125 ml/m^3^.

Ausgehend von der LOAEC von 100 ml/m^3^ der Wirkung auf den Atemtrakt, wird unter Berücksichtigung der geringen Ausprägung des Effekts (nur bei elektronenmikroskopischer Untersuchung) auf eine NAEC extrapoliert (1:2). Histologische Befunde an der Nase wurden mit üblichen Methoden bei weit höheren Konzentrationen an Ratten und Mäusen nicht beobachtet, deshalb wird auf den Faktor für die Speziesübertragung verzichtet und unter Berücksichtigung des erhöhten Atemvolumens (weil die Trachea betroffen ist und nicht nur die Nase, 1:2) und des Preferred-Value-Approach ein MAK-Wert von 20 ml Tetrahydrofuran/m^3^ (60 mg/m^3^) festgelegt. Dieser MAK-Wert schützt auch vor den systemischen Wirkungen.

**Spitzenbegrenzung. **Da der MAK-Wert auf einem lokalen Effekt beruht, wird Tetrahydrofuran weiterhin in die Kurzzeitwert-Kategorie I eingruppiert. Der bisherige Überschreitungsfaktor von 2 wird beibehalten, da zwar keine gezielte Studie zu sensorischen Reizeffekten vorliegt, aber in den toxikokinetischen Studien die Probanden drei Stunden bis zu 200 ml/m^3^ exponiert waren, also in diesem Bereich keine deutlichen Reizwirkungen aufgetreten sein dürften. 

**Fruchtschädigende Wirkung. **In einer Studie zur pränatalen Entwicklungstoxizität nach OECD-Prüfrichtlinie 414 an Sprague-Dawley-Ratten mit täglicher Ganzkörperexposition an sechs Stunden pro Tag trat bei 5060 ml/m^3^ bei gleichzeitiger Maternaltoxizität (verringerte Körpergewichtszunahme) ein reduziertes Fetengewicht auf (Greim 2003; Mast et al. 1992). Eine weitere Studie zur pränatalen Entwicklungstoxizität ähnlich der OECD-Prüfrichtlinie 414 an Crl:CD-Ratten mit täglicher Ganzkörperexposition an sechs Stunden pro Tag erbrachte bei den Feten bei 4934 ml/m^3^ erniedrigte Körpergewichte und eine verzögerte Ossifikation der Sternebrae. Die Muttertiere wiesen dabei erhebliche toxische Effekte wie erniedrigte Körpergewichtszunahme und Futteraufnahme, Lethargie und taumelnden Gang auf (Haskell Laboratories 1982). Bei Swiss-CD1-Mäusen war in einer Ganzkörperexpositionsstudie mit täglicher Exposition an sechs Stunden pro Tag zur pränatalen Entwicklungstoxizität nach OECD-Prüfrichtlinie 414 bei 1800 ml/m^3^ die Anzahl lebender Feten/Wurf bei gleichzeitiger maternaler Toxizität (Sedierung, erniedrigtes Uterusgewicht) verringert (9,3 ± 4,4; Kontrolle: 11,9 ± 1,9).

Weder bei Ratten noch bei Mäusen wurden teratogene Effekte beobachtet (Greim 2003; Haskell Laboratories 1980, 1982; Mast et al. 1992).

In einer 2-Generationenstudie nach OECD-Prüfrichtlinie 416 kam es bis zur höchsten Dosierung von 790 mg/kg KG und Tag bei gleichzeitiger Maternaltoxizität (erniedrigte Futteraufnahme und Körpergewichtszunahme, erhöhtes relatives Nierengewicht) bis zum 4. Postnataltag nicht zu Effekten auf die Nachkommen (siehe Abschnitt 5.5.1; BASF AG 1994; Greim 2003; Hellwig et al. 2002). Daraus leitet sich ein NOAEL für Perinataltoxizität von 790 mg/kg KG und Tag ab. Die bewertungsrelevanten NOAEC/NOAEL für Entwicklungs- und Perinataltoxizität und deren toxikokinetische Übertragung in eine Konzentration in der Luft am Arbeitsplatz sind in Tabelle 3 dargestellt.

**Tab.3:** Bewertungsrelevante NOAEC/NOAEL für Ratte und Maus bei oralen Studien einschließlich der toxikokinetischen Umrechnung in eine Luftkonzentration und die Abstände zum MAK-Wert von 20 ml/m^3^ (60 mg/m^3^)

Literatur	Exposition	NOAEC/NOAEL: Endpunkt	Toxikokinetische Umrechnung	Abstand zum MAK-Wert
Ratte				
Greim 2003; Mast et al. 1992	Pränatal, Inhalation, Ganzkörper	1830 ml/m^3^: Entwicklungstoxizität	915 ml/m^3^^a)^	46-fach
Haskell Laboratories 1980, 1982	Pränatal, Inhalation, Ganzkörper	1002 ml/m^3^: Entwicklungstoxizität	501 ml/m^3^^a)^	25-fach
BASF AG 1996; Hellwig et al. 2002	Prä- u. postnatal, Trinkwasser	790 mg/kg KG u. d (höchste Dosis): Perinataltoxizität	2652 mg/m^3^^b)^	44-fach
Maus				
Greim 2003; Mast et al. 1992	Pränatal, Inhalation, Ganzkörper	598 ml/m^3^: Entwicklungstoxizität	300 ml/m^3^^a)^	15-fach

a) Berücksichtigung des erhöhten Atemvolumens (1:2)

b) 1/4 (Ratte) **×** 70 kg/10 m 3 **×** orale Resorption 100 % (experimentell fast vollständig bei Ratte und Maus, DuPont 1998) / inhalative Resorption 73 % (Teramoto et al. 1988), Umrechnung von 7 auf 5 Tage pro Woche

Die Abstände der berechneten NOAEC für Entwicklungs- und Perinataltoxizität sind ausreichend groß, und die bisherige Zuordnung von Tetrahydrofuran zur Schwangerschaftsgruppe C wird bestätigt.

**Krebserzeugende Wirkung.  **Nach Inhalation werden in einer chronischen Studie nicht statistisch signifikant erhöhte Inzidenzen von Nierenadenomen bei männlichen F344-Ratten und statistisch signifikant erhöhte Inzidenzen von Leberadenomen und -karzinomen bei weiblichen B6C3F1-Mäusen bei einer Konzentration von 1800 ml Tetrahydrofuran/m^3^ festgestellt. 

Es ist keine genotoxische Wirkung für Tetrahydrofuran gezeigt, sodass dies als Ursache für eine Tumorentstehung ausgeschlossen wird, ebenso wie Zytotoxizität. Die Lebertumoren bei weiblichen Mäusen stehen mechanistisch mit einer Aktivierung des CAR/PXR-Rezeptors und der daraus resultierenden Zellproliferation in Zusammenhang. Die Aktivierung des CAR/PXR-Rezeptors ist mit Tetrahydrofuran im Gegensatz zum bekannten Tumorpromotor Phenobarbital nur schwach ausgeprägt (siehe Abschnitt 2). Zudem treten statistisch signifikant erhöhte Inzidenzen von Leberkarzinomen im suszeptiblen Mausstamm B6C3F1 nur bei weiblichen Tieren bei der höchsten Konzentration von 1800 ml Tetrahydrofuran/m^3^ auf, die bei männlichen Tieren bereits aufgrund toxischer Wirkung eine erhöhte Mortalität zur Folge hatte. 

Die Nierenadenome entwickelten sich nur bei männlichen Ratten. Mehrere Studien liefern Hinweise dafür, dass die zu Grunde liegenden Mechanismen α-2u-Globulin- und CPN-assoziiert sind. Beide Mechanismen sind nicht humanrelevant (siehe Abschnitt 2). Deshalb wird Tetrahydrofuran nicht mehr in eine Kanzerogenitäts-Kategorie eingestuft.

**Keimzellmutagene Wirkung.  **Tetrahydrofuran ist nicht genotoxisch und wird daher nicht in eine Kategorie für Keimzellmutagene eingestuft.

**Hautresorption.  **Die dermale Aufnahme über die Gasphase ist maximal 5,9 % (Brooke et al. 1998) der Gesamtaufnahme über Haut und Lunge und somit vernachlässigbar. 

Aus einer In-vitro-Studie mit Humanhaut ergibt sich eine Aufnahme von 2720 mg unter Standardbedingungen für 10%iges wässriges Tetrahydrofuran. Die systemische NOAEC ist 500 ml Tetrahydrofuran/m^3^ (1500 mg/m^3^) für narkotische Wirkung in einer 13-Wochen-Studie an Ratten. Die berechnete NAEC für systemische Wirkung beim Menschen am Arbeitsplatz ist 125 ml/m^3^ (siehe MAK-Wert). Mit einem Atemvolumen in acht Stunden von 10 m^3^ und einer inhalativen Resorption von 73 % (Abschnitt 3.1) errechnet sich eine systemisch tolerable Menge von 2738 mg. Die dermale Aufnahme von wässrigem Tetrahydrofuran beträgt mehr als 25 % der systemisch tolerablen Menge, daher bleibt Tetrahydrofuran mit „H“ markiert.

**Sensibilisierende Wirkung.  **Zur Hautsensibilisierung liegen keine bewertbaren Befunde am Menschen sowie nur negative tierexperimentelle Daten vor. Für Tetrahydrofuran erfolgt daher keine Markierung mit „Sh“. Zur Atemwegssensibilisierung liegen keine Daten vor. Daher erfolgt keine Markierung mit „Sa“.
